# Expansion and evolutionary patterns of cysteine-rich peptides in plants

**DOI:** 10.1186/s12864-017-3948-3

**Published:** 2017-08-14

**Authors:** Xing Liu, Huping Zhang, Huijun Jiao, Leiting Li, Xin Qiao, Musana Rwalinda Fabrice, Juyou Wu, Shaoling Zhang

**Affiliations:** Center of Pear Engineering Technology Research, State Key Laboratory of Crop Genetics and Germplasm Enhancement, College of Horticulture, Nanjing Agricultural University, Nanjing, 210095 China

**Keywords:** Cysteine-rich peptide, Expression divergence, Clustered genes, Divergent evolution pattern, Gene duplication, Positive selection, Self-incompatibility

## Abstract

**Background:**

Cysteine-rich peptides (CRPs) are gaining recognition as regulators of cell–cell communication in plants.

**Results:**

We identified 9556 CRPs in 12 plant species and analysed their evolutionary patterns. In most angiosperm plants, whole genome duplication and segmental duplication are the major factors driving the expansion of CRP family member genes, especially signal peptides. About 30% of the CRP genes were found clustered on the chromosomes, except in maize (*Zea mays*). Considerable collinearities between CRP genes between or within species reveal several syntenic regions on the chromosomes. Different subfamilies display diverse evolutionary rates, suggesting that these subfamilies are subjected to different selective pressures. CRPs in different duplication models also show contrasting evolutionary rates, although the underlying mechanism is unclear because of the complexity of gene evolution. The 1281 positively selected genes identified are probably generated within a certain period of time. While most of these belonged to maize and sorghum (*Sorghum bicolor*), new CRP functions would also be expected. Up-regulation of 10 CRPs was observed in self-pollinated pear pistils and pollen tubes under self S-RNase treatments in vitro. The expression divergence between different CRP gene duplication types suggests that different duplication mechanisms affected the fate of the duplicated CRPs.

**Conclusion:**

Our analyses of the evolution of the CRP gene family provides a unique view of the evolution of this large gene family.

**Electronic supplementary material:**

The online version of this article (doi:10.1186/s12864-017-3948-3) contains supplementary material, which is available to authorized users.

## Background

Cysteine-rich peptides (CRPs) are a group of proteins that mediate many aspects of plant physiology and development. These proteins have emerged as plant peptide ligands that trigger membrane receptors to induce plant growth, plant defence, plant–bacteria symbiosis and plant reproduction [1, 2]. The first CRP to be elucidated is systemin, which induces over 15 defensive genes [3]. Rapid alkalinisation factor (RALF) has been identified in many crops as a signalling peptide that causes alkalinisation of the culture medium [4–6]. A further study revealed that RALF induces rapid activation of MAP kinases [7]. Members of the epidermal patterning factor family, each of which contains eight conserved cysteines, have been found to regulate stomata formation [8–10]. Antimicrobial peptides belonging to a large family of nodule-specific CRPs form disulphide bridges and function in nodule development [11, 12]. Self-incompatibility (SI) is a mechanism in flowering plants that prevents inbreeding. Many CRPs participate in this process, including SI determinants in pistil S-locus glycoproteins, the S-locus receptor-like kinase, the *Papaver rhoeas* stigma S-determinant and the pollen S-determinant (PrpS) [13–18]. Pollen tube growth and guidance are also controlled by CRPs. Lat52 regulates pollen germination and binds LePRK2, while its family members bind two different CRPs [19, 20]. CRPs such as LURE are considered to be involved in the mechanism whereby the pollen tube is attracted to synergid cells once sensed by the pollen receptor-like kinase [21]. Finally, egg cell 1 is a signal peptide that regulates exocytosis and sperm plasma membrane modifications by interacting with gametes [22].

In addition to the elucidation of this wide range of plant physiological and developmental processes regulated by CRPs, pioneering work on genome-wide identification of CRPs has been presented for several species [23, 24]. Numerous CRPs have been exhaustively identified and classified into divergent subfamilies using an iterative motif searching method. Some CRP expression patterns in maize (*Zea mays*) and different accessions of barrelclover (*Medicago truncatula*) have also been revealed [25, 26].

While genome-wide analyses of CRPs have increased our understanding of this family, the patterns of CRP gene evolution have not been identified. The abundant CRP gene family clearly has divergent evolutionary mechanisms and functional relevance. The increasing number of sequenced genomes now allows for the gathering of CRP gene sequences from various species to study evolutionary patterns and duplication events.

This study aims to identify CRP genes and investigate genome organisation, gene duplication types, family evolutionary patterns and expression patterns in the genes of developing fruits and pollen of the pear *Pyrus bretschneideri*. Considering the large number of CRPs and their divergent characteristics, the goal of this work is not only to provide a further understanding of the CRP gene family but also to investigate plant genome expansion and characteristics of gene evolution.

## Methods

### Identification of CRPs

Our search strategy was based on successive iterations of hidden Markov model (HMM) builds and BlastP similarity searches of CRPs in 12 species [27]. We then used the HMMs generated by 516 CRPs to search all of the predicted pear peptides [28]. These motif models were constructed only from mature CRP peptides included in the comprehensive UniProt protein dataset, The Institute for Genomic Research 33 plant gene indices and the entire genome of rockcress (*Arabidopsis* spp.) and rice (*Oryza* spp.) [29]. After we filtered these results using an E value <0.01, 385 annotated CRPs were confirmed. All of these peptides were scanned with the SignalP4.0 program to examine their signal peptides.

Small Peptide Alignment Discovery Application (SPADA) was used to search for CRPs that were not found by HMMER. SPADA translated the genome fasta sequences into all six reading frames, and the open reading frames were extracted. A Hmmsearch using family-specific HMM then was performed [30].

### Duplication of CRP genes

All CRP collinearity analyses between and within species were carried out using MCScanX [31]. Collinear alignments were retained for an E value <0.01. Circos was used to illustrate the distribution of CRP genes on the chromosome and the collinear relationships [32].

### Selection pressure and evolution rate

Calculation of the Ka/Ks value was accomplished using the KaKs_Calculator 2.0, while the method for estimating Ka and Ks followed the procedure of Nei and Gojobori (1986) [33, 34]. The gene and peptide files were converted to AXT format using a perl script to calculate Ka/Ks values. The generated Ka/Ks values were filtered by *P* value (*P* < 0.05). The selected sites were estimated by codeml in PAML and site model including M0, M7, M8 were selected for the evaluated Likelihood ratio test.

### Real-time quantitative PCR (qRT-PCR)

qRT-PCR was performed using the LightCycler 480 SYBR GREEN I Master (Roche, USA). Each reaction mixture (20 μL) contained 10 μL of LightCycler 480 SYBR GREEN I Master, 6 μL of nuclease-free water, 1 μL of each primer and 2 μL of diluted cDNA. All reactions were repeated in triplicate. The qRT-PCR reaction conditions were as follows: pre-incubation at 95 °C for 5 min, 55 cycles of 94 °C for 5 s, 60 °C for 30 s, 72 °C for 30 s, extension at 72 °C for 3 min and fluorescence data collection at 60 °C. The average threshold cycle (Ct) per sample was calculated. The relative expression was determined using the 2-△△CT method using *Pyrus* Actin (AF386514) as the internal control gene in pollen and pollinated pistils. All primers are shown in Additional file 1: Table S4. The reads per kilobase per million mappeds (RPKM) of Pbr032147.1, Pbr040311.1 and Pbr040304.1 in mature pollen were standardisation to give the corresponding relative expression in mature pollen.

### Gene expression pattern

To examine the expression of CRP genes, pear fruit samples at 15, 36, 80, 110, 145 and 167 days after flowering were used. Three or more fruits were collected at each stage from the Nanjing Agricultural University experimental farm in 2011. The RPKMs for pollens and pollen tubes were acquired in our previous studies [35, 36].

### Preparation of S-RNase and pollen tube cultures

Using the isolation method described in our previous report [37], we prepared 4 g of ‘Fengshui’ styles. The pollens of ‘Dangshangsuli’ and ‘Fengshui’ were cultured in darkness and in a basal medium. The basal medium consisted of 0.03 M MES, 0.03% Ca(NO_3_)_2_, 0.01% boric acid and 10% sucrose. The pH was adjusted to 6.2 with KOH. S-RNase was added to the medium after 40 min of preculturing.

## Results

### Collection and identification of CRP in 12 species

To reveal the universal plant CRP characteristics, our analysis focused on CRPs in 12 species: pear, mei (*Prunus mume*), strawberry (*Fragaria vesca*), cabbage (*Brassica rapa*), black cottonwood (*Populous trichocarpa*), soybean (*Glycine max*), peach (*Prunus persica*), grape (*Vitis vinfera*), tomato (*Solanum lycopersicum*), maize, sorghum (*Sorghum bicolor*) and green alga (*Volvox carteri*). A total of 4210 CRPs were collected from these species and identified using HMMER 3.0 (Additional file 2: Table S1), using HMM builds identified previously. Previous studies suggest that popular search tools fail to collect most CRPs because of their highly divergent sequences [30]. A new search tool, SPADA, was used to collect 5346 CRPs. Finally, a total of 9556 CRPs was identified. CRPs identified using SPADA constituted 37.66% to 66.85% of the total (Table 1). The green alga contained the smallest number of CRPs compared with other higher plants, both in the number and percentage of total protein-coding genes (Table 1).

**Table 1:** Number of CRP genes in 12 different species

Species	CRPs identified from proteome	CRPs identified by SPADA	Total CRPs identified	CRPs identified by SPADA/Total CRPs	Genome size (Mb)	Chromosome number
Sorghum	327	509	836	60.89%	211	20
Mei	305	279	584	47.77%	280	8
Peach	294	355	649	54.70%	227.3	8
Strawberry	251	267	518	51.54%	240	7
Tomato	385	490	875	56.00%	900	12
Poplar	420	472	892	52.91%	422.9	19
Cabbage	560	1010	1570	64.33%	283.3	10
Grape	180	363	543	66.85%	487	19
Soybean	583	673	1256	53.58%	978	20
Maize	472	498	970	51.34%	2300	10
Volvox	48	29	77	37.66%	131.2	-
Pear	385	401	786	51.02%	512	17

**Fig. 1:**
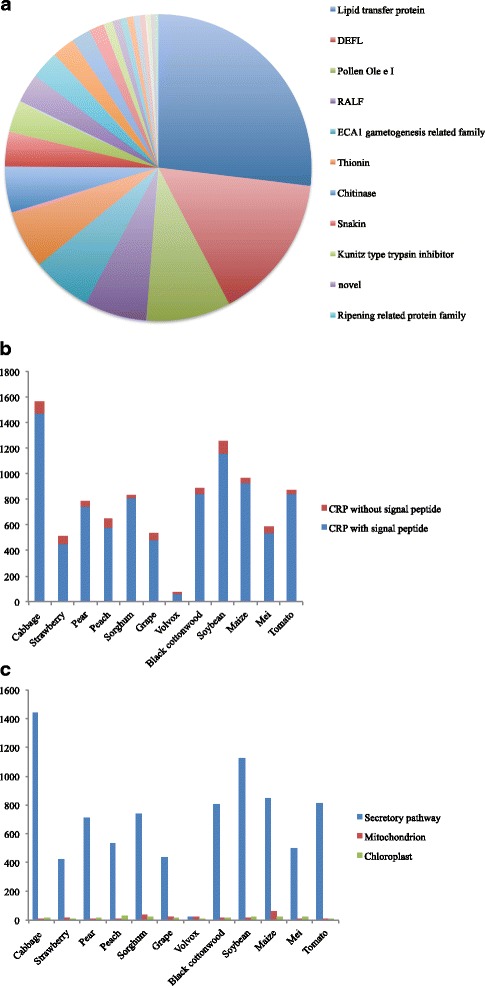
CRP subfamilies and their species-specific signal peptides. **a** Subfamily composition in all CRPs; (**b**) Comparison of CRPs with and without signal peptides; (**c**) Distribution of the different signal peptides

The 9556 CRPs identified here were classified into 32 subfamilies (Fig. 1a). The largest subfamily was that of the lipid transfer proteins, which contained 2560 members. The smallest subfamily, Gamma gliadin, contained only a single CRP. CRPs with N-terminal signal peptides constituted the majority of identified CRPs in each species except green algae. Many CRPs contained one signal peptide, most of which were secretory pathway signals (Fig. 1b). The Cys residue in different subfamilies showed divergent distributions that are directly related to their functions (Additional file 3: Figure S1).

### Expansion pattern and collinearity of CRPs in angiosperm plants

**Fig. 2:**
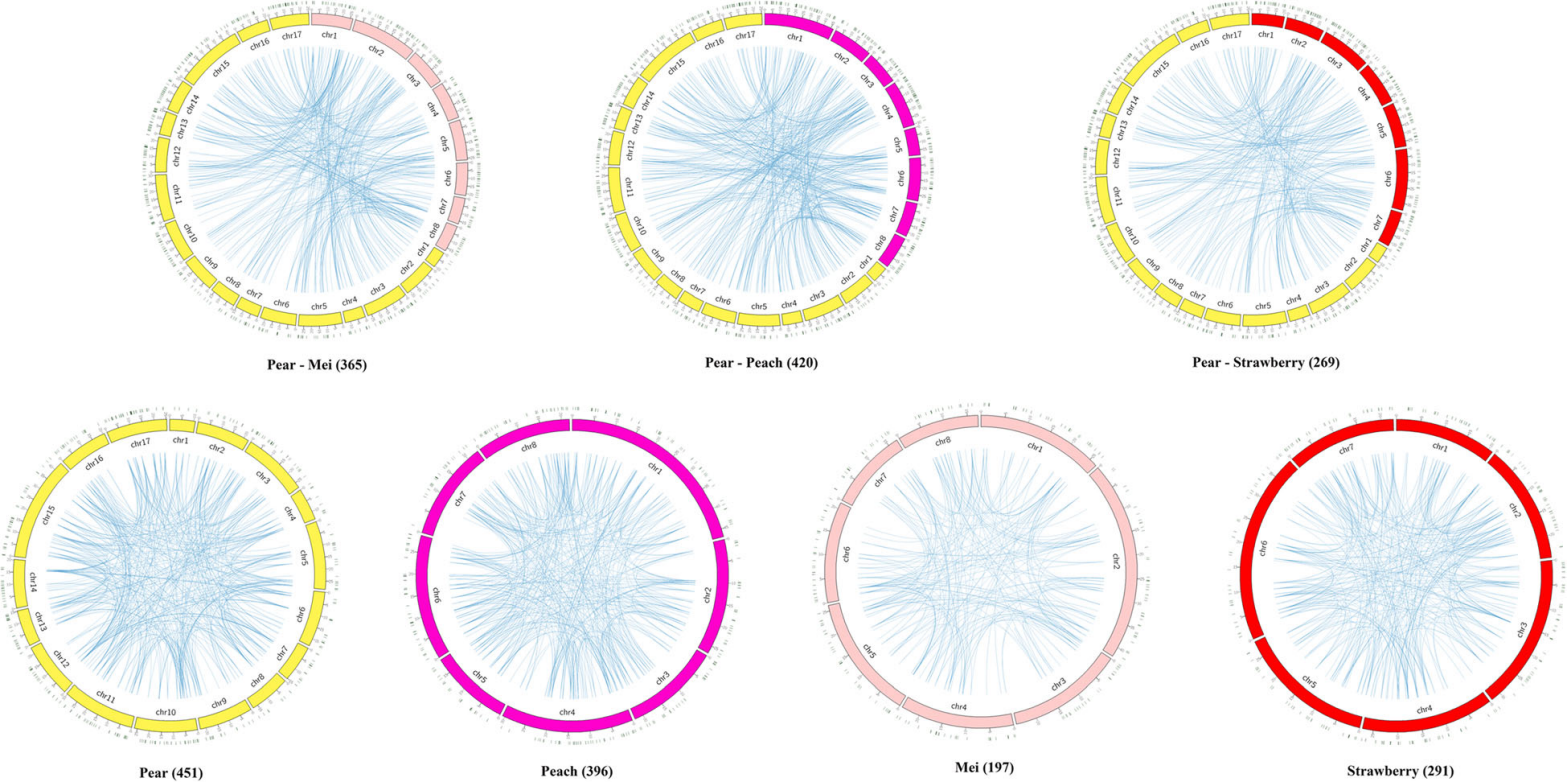
Collinearity of CRPs in the Rosaceae. The blue lines across the chromosomes indicate collinearity between CRP gene pairs. Lines above the chromosomes indicate CRP genes. Numbers in parentheses indicate the number of collinear gene pairs. Different coloured chromosomes indicate different species: *yellow*, *pear*; *maroon*, *peach*; *pink*, *mei* and *red*, *strawberry*

To determine how CRPs duplicate, the patterns of collinearity between and within species were investigated. Most CRPs in our analysis could be assigned to chromosomes, except for those of green algae (Additional file 4: Table S2). The other 11 genomes were analysed using MCScanX to determine collinearity alignments of all genes within a species (Additional file 5: Figure S2). Considering the adjacent genetic relationships between peach, pear, mei, strawberry and all members of the Rosaceae, we conducted collinearity alignment analysis for these four species (Fig. 2). Based on the number of CRPs within a species, these collinear CRP gene pairs were not linearly relational. Further, the chromosome number and size did not affect the number of duplicated gene pairs, as shown in black cottonwood and grape (Additional file 5: Figure S2). The number of collinear gene pairs differed between these species, despite similar numbers and sizes of chromosomes.

A gene cluster is a group of two or more genes from the same family located within a few thousand base pairs (bp) of each other. As genes in eukaryotic genomes are often clustered, we counted the CRP clusters, defining a cluster as two or more CRP genes with genomic distances of less than 10, 000 bp (Table 2). Our results show that 22–39% of CRPs in most species were clustered, except in maize, in which only 7.32% of CRPs were clustered. The most significant difference between maize and the other species was the larger percentage of transposons in the genome of the former, even compared with sorghum, a closely-related species. During the evolutionary divergence of maize from sorghum, an additional whole genome duplication (WGD) event occurred about 5–12 million years ago, and the expansion of long terminal repeat retrotransposons (LTR) enlarged the maize genome to 2.3 gigabases. Considering that many species had undergone more than one round of WGD, the proliferation of LTRs might have disrupted clustered CRPs.

**Table 2:** CRP gene clusters in plants

Species	Cluster number	Cluster members >2	Cluster genes	Cluster genes/total CRP genes
Sorghum	103	27	212	25.36%
Mei	74	31	189	32.36%
Peach	71	28	171	26.35%
Strawberry	73	15	203	39.19%
Tomato	113	35	316	36.11%
Poplar	103	42	351	39.35%
Cabbage	179	51	415	26.43%
Grape	47	15	137	25.23%
Soybean	102	12	287	22.85%
Maize	31	7	71	7.32%
Pear	84	35	303	38.55%

To better comprehend the impact of WGD and fractionation on the size of the CRP family, duplication models for these genes were generated using MCScanX (Additional file 6: Figure S3). WGD was the major type of CRP duplication in all species studied except maize and volvox, suggesting that many CRPs were retained after WGD in plants. In maize, which also experienced several rounds of WGD, most CRPs were generated by single gene duplication, possibly influenced by the frequent activity of transposons. The CRP copy number in volvox, which underwent no recent WGD, is lower than that of CRPs from other plants. These results suggest that WGD is the major force in CRP gene family expansion.

### Evolutionary patterns and selection pressures

The large number of CRPs in plant genomes allows for characterisation of their genetic evolution. The Ka and Ks of gene pairs in different subfamilies were deduced using the KaKs_Calculator 2.0. This determination is the first step towards estimating the evolutionary rate between divergent sequences within different subfamilies. Twelve subfamilies were selected for this analysis, yielding more than 1000 valid gene pairs after removing null values. All subfamilies showed a conserved evolutionary rate, with Ka/Ks values all lower than 0.5 (Additional file 7: Figure S4). However, some families were found to have more than one peak, including Pollen Ole e I, Kunitz type trypsin inhibitor and ECA gametogenesis related family. The three peaks of Pollen Ole I indicate that there were three evolutionary orientations for subfamily members. We infer that the different evolutionary pressures acting on the subfamily members might account for the divergent roles they played.

**Fig. 3:**
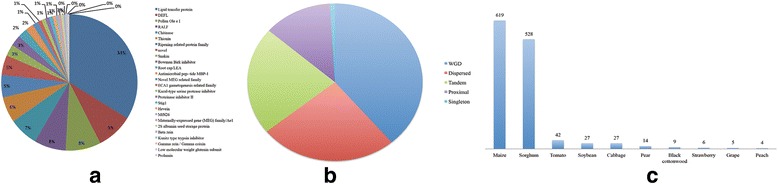
Comparison of CRP genes under positive selection. **a** CRP genes under positive selection in different subfamilies; (**b**) CRP genes under positive selection in different duplication types; the percentage of the corresponding total CRP genes of different duplication types are in parenthesis; (**c**) Number of CRP genes under positive selection by species

Genes under positive selection are usually expected to generate new functions. A total of 1281 CRP genes, including 26 subfamilies and 10 species, were estimated to be under positive selection (Fig. 3a, c). The lipid transfer protein subfamily possessed not only the largest number of CRPs but also the most divergent evolutionary pattern (Fig. 3a). For example, CRP genes under positive selection encompassed five duplication models and CRP genes generated by WGD played a major role (Fig. 3b). In addition, the number of CRP genes under positive selection was unequal in different species (Fig. 3c). An unusual finding was that the CRP genes under positive selection overwhelmingly came from two domesticated monocots, sorghum and maize (Fig. 3c). Whether the number of CRP genes under positive selection was affected by artificial selection or the complexity of the genome is unclear and requires further study.

**Fig. 4:**
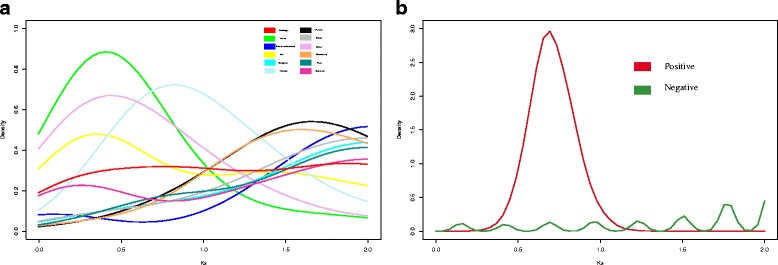
Comparison of CRP gene Ks values. **a** Comparison of CRP gene Ks values in 11 angiosperm plants; (**b**) Comparison of Ks values between positively and negatively selected genes

The divergence times for CRP genes should be compared to further clarify their evolutionary patterns. Given that genome rearrangement usually accelerates the evolutionary rate, different plant genomes do not always occur at a common rate; thus, the estimation of accurate divergence times was not feasible in our analysis. Ks is less affected by selection and is usually regarded as an appropriate proxy for estimating the evolutionary divergence time [38]. Given the complexity of the process of gene duplication during evolution, it is difficult to analyse duplications that occurred a long time ago. Thus, we chose duplicated CRP gene pairs in different species with a Ks value <2 for estimating the occurrence of duplication events (Fig. 4a). Results show that the Ks of nearly all angiosperms had at least one peak, during which the duplicated gene is considered to have emerged. The Ks of soybean duplicated CRP genes showed no obvious peak, suggesting that the duplication of CRP genes in soybean occurred in a non-explosive manner, similar to that in cabbage.

Based on the density distribution of Ks, we speculate that duplicated CRP genes arose by multiple means, including WGD, segmental duplication and single gene duplication. Some species, including pear, maize, sorghum and grape, underwent WGD or segmental duplication to expand the CRP gene family. Other species, including mei, peach and strawberry, underwent only single gene duplication. Soybean, cabbage, black cottonwood and tomato genomes underwent both WGD and single gene duplication to expand the CRP gene family.

To explore the occurrence of positive and negative selection of CRP genes, we estimated the relative divergence times between them. We found that positively selected genes had a peak range from 0.5–1.0, while negatively selected genes had a smaller peak, near 2.0 (Fig. 4b). Clearly, the explosive expansion of CRP genes under positive selection was concentrated in a certain time period. However, because the speed of angiosperm molecular clocks differ, it was difficult to determine when positive selection acted on the coding sequences of CRP genes.

**Fig. 5:**
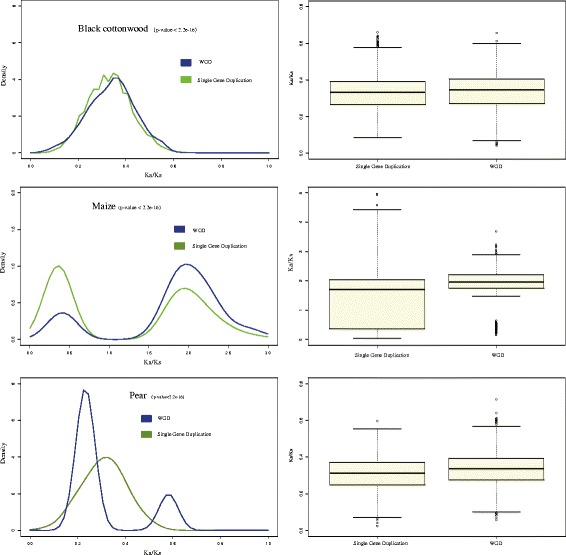
Comparison of evolutionary rates between CRP duplicates arising from WGD or segmental duplications and those arising from single gene duplications in *pear*, *black cottonwood* and *maize*; (**a**) *pear*, (**b**) *black cottonwood*, (**c**) *maize*

Because a number of CRP genes arose from WGD or segmental duplication, the fate of these duplicated genes should be clarified. Thus, to compare the evolutionary rates between the different types of CRP gene duplications, we estimated the Ka/Ks values for the different duplication models in each species. Because the number of CRP genes of each duplication type differs between species, only three species (maize, pear and black cottonwood) were included in our determination of evolutionary rates. We chose these three species because each contains a sufficient number of both duplication types. In our analysis, duplicates from single gene duplications included tandem duplicates, proximal duplicates and dispersed duplicates. Our results show that in all three species, CRP duplicates from WGD and segmental duplication displayed a significantly faster evolutionary rate than did duplicates from single gene duplication (Fig. 5). In maize, both types of duplicates included positively and negatively selected genes; the Ka/Ks was >2 for most of the genes under positive selection, suggesting that the positive selection was strong (Fig. 5). Only the Ka/Ks density peak for black cottonwood and pear indicated that genes of different duplication types evolved at the same rate under purifying selection (Fig. 5). To better elucidate the relationship between evolutionary pattern and duplication type, we investigated peach, mei and strawberry, which share a substantial number of single gene duplication types, including tandem duplication, proximal duplication and dispersed duplication. We compared the Ka/Ks values between different duplication types to estimate the evolutionary rate of a different single gene duplication type. Although evolutionary rate of dispersed duplication was higher in mei and strawberry, results of the Bartlett test indicated no significant difference between the three groups (Additional file 8: Figure S5). This result implies that duplications generated by WGD and segmental duplication evolved faster than did those from single gene duplications and that evolutionary orientation was not associated with duplication type.

The pear CRP genes *Pbr040304.1*, *Pbr040311.1* and *Pbr032147.1* were observed to be positively selected by pairwise comparison using the KaKs_Calculator 2.0. We performed BlastP analysis to acquire the homologies of the three positively selected CRPs. Eight closely-related homologous genes were selected to construct a Maximum Likelihood tree for the three CRP genes using RAxML. The codeml in PAML was used to estimate the selection pressure acting on genes in this tree. Of the 1834 aligned amino acid sites, 287 were determined to be positively selected, while 21 amino acid sites were determined to be significantly positively selected (Additional file 9: Table S3). Three models were selected to estimate the selection pressure, and the Likelihood ratio test suggested that the determination of the positively selected sites was reliable (Table 3).

**Table 3:** Parameter estimates and Likelihood scores under models of variable ω among sites of positively selected pear CRP genes

Nested Model	Ns sites	dN/dS	−*ln* L	Likelihood ratio test
M0: One-ratio	0	1.12196	15,044.66039	Two-ratio vs. one-ratio: *P* < 0.01
M7: beta	7	1	15,046.51687	
M8: beta&ω	8	3.53064	14,963.64578	

**Fig. 6:**
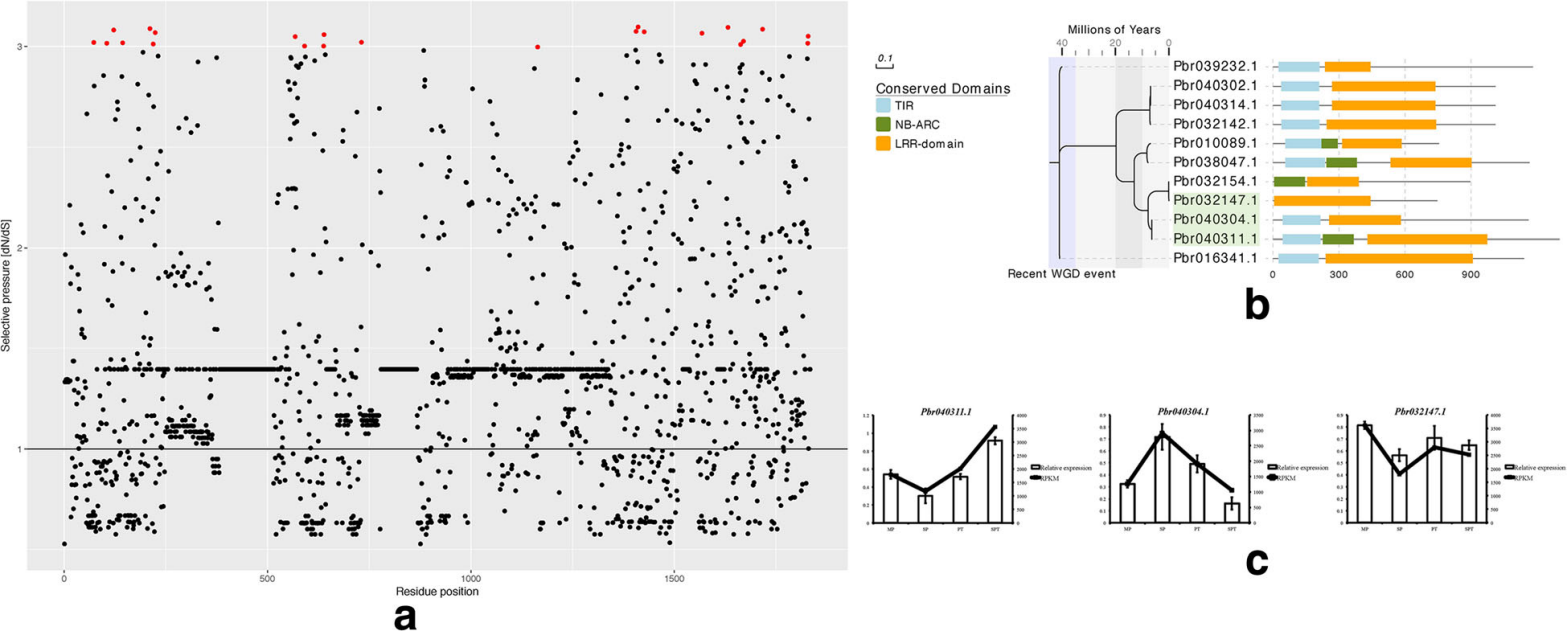
Analysis of positively selected CRP genes in pear. **a** Distribution of estimated amino acid sites; (**b**) Maximum Likelihood phylogenetic tree constructed using three positively selected CRP genes and eight closely-related homologous genes and their corresponding conserved domains; green, three pear CRP genes; violet, recent WGD events; (**c**) Expression pattern of three positively selected CRP genes during pear pollen development. HP, hydrated pollen; MP, mature pollen; PT, hydrated pollen tube; SPT, stopped pollen tube

Most of the dN/dS values for the investigated sites were >1, and most of the significant values were >3 (Fig. 6a). The time of divergence of the three positively selected pear CRP genes was found to be quite close to the recent WGD event that generated most of the CRP genes. During the period 5–10 million years ago, Pbr040304.1 and Pbr040311.1 were divided, while Pbr040304.1 lacked an NB-ARC domain (PF00931). The divergence of Pbr032147.1 and Pbr032154.1 was quite close to that time period, and Pbr032154.1 lacked a TIR domain (PF1582). However, Pbr032147.1 was the only member of this tree to retain the LRR-domain (PF00560). According to their phylogenetic relationship, we speculate that the ancestor of *Pbr032154.1*, *Pbr032147.1*, *Pbr040304.1* and *Pbr040311.1* was a CRP until Pbr032154.1 lost the characteristics of CRP. In particular, *Pbr040311.1* and *Pbr040304.1,* both of which were duplicated gene pairs, showed opposite expression patterns during the stages of pollen development. This trend was confirmed by the qPCR results.

### CRP expression divergence in pear

The expression patterns of CRPs in pear were investigated using the RPKMs of two representative organs. The fruit and pollen of pear in different developmental stages were investigated via transcriptome sequencing using their expression profiles. A total of 207 CRPs were expressed in six fruit development stages, and 385 CRPs were expressed in four pollen stages (Additional file 10: Figure S6, Additional file 11: Figure S7). Genes in the same cluster displayed similar expression patterns. The CRP expression patterns in fruit development exhibited six distinct clusters, while only four distinct clusters were exhibited in pollen. The expression patterns in each distinct CRP pollen cluster were more consistent than those in fruit development. The CRP expression patterns in pear suggest that a vegetative organ has a more divergent CRP expression pattern than does a reproductive organ, while the reproductive organ has more CRP members to regulate biological processes.

To determine the influence of gene duplication type on expression divergence, we explored the relationship between duplication type and gene expression. Our collinearity analysis revealed that large-scale gene duplication events created duplicated CRPs. Given that gene duplication can affect the expression patterns of the duplicated genes [39], we performed correlation analysis of CRP expression and their duplication modes. In the expression profiles during pollen and pollen tube development, the expression divergence trends were similar; the exception was duplicated proximal CRPs, which were less divergent than the other types of duplicated CRPs (Additional file 8: Figure S8). Numerous studies have treated proximal duplication as a form of tandem duplication, even though the genetic mechanisms underlying these duplications differ. Our analysis reveals that CRPs from tandem duplications correlate higher with gene expression than do CRPs from proximal duplications, suggesting that these two similar types of duplication affect gene expression in different ways. Recent studies have also found that proximally duplicated genes have a different evolutionary fate and features than do tandem and dispersed duplicated genes [40].

Another dynamic developmental expression profile in fruits demonstrated an expression divergence trend that differed between the CRP duplication types: Singleton > Tandem > WGD or Segmental ≈ Proximal > Dispersed (Additional file 12: Figure S8a). The singleton CRPs, which showed the highest level of expression divergence, might account for the distinct evolutionary consequences. Considering that tandem and block duplication creates a large number of duplicated genes commonly associated with speciation [41], a higher level of expression divergence could assist plants in their adaptation to new environments during speciation.

We also found that CRP expression divergence across different profiles was distinct. For example, the expression of CRPs during fruit development was much more divergent than that of CRPs expressed during pollen and pollen tube development (Additional file 12: Figure S8). This observation suggests that CRPs play more divergent roles during fruit development than during pollen and pollen tube growth.

### CRP expression patterns respond to self-incompatibility in pear

Many studies report that CRPs play vital roles in pollen development and self-incompatibility in Arabidopsis, cabbage and tobacco [16, 42, 43]. Fewer reports address whether CRPs function during self-incompatibility processes in Rosaceae plants. Our RNA-seq results indicate that pistils pollinated with self and non-self-pollen exhibit different mechanisms involving different genes. Ten CRP genes in the two libraries presented similar trends, with up-regulation of CRP genes in self-incompatible pistils (Fig. 6b). All 10 of these CRPs belong to Pollen Ole I or DEFL, and 3 conserved Cys residues were shared by all 10 CRPs (Fig. 6a).

**Fig. 7:**
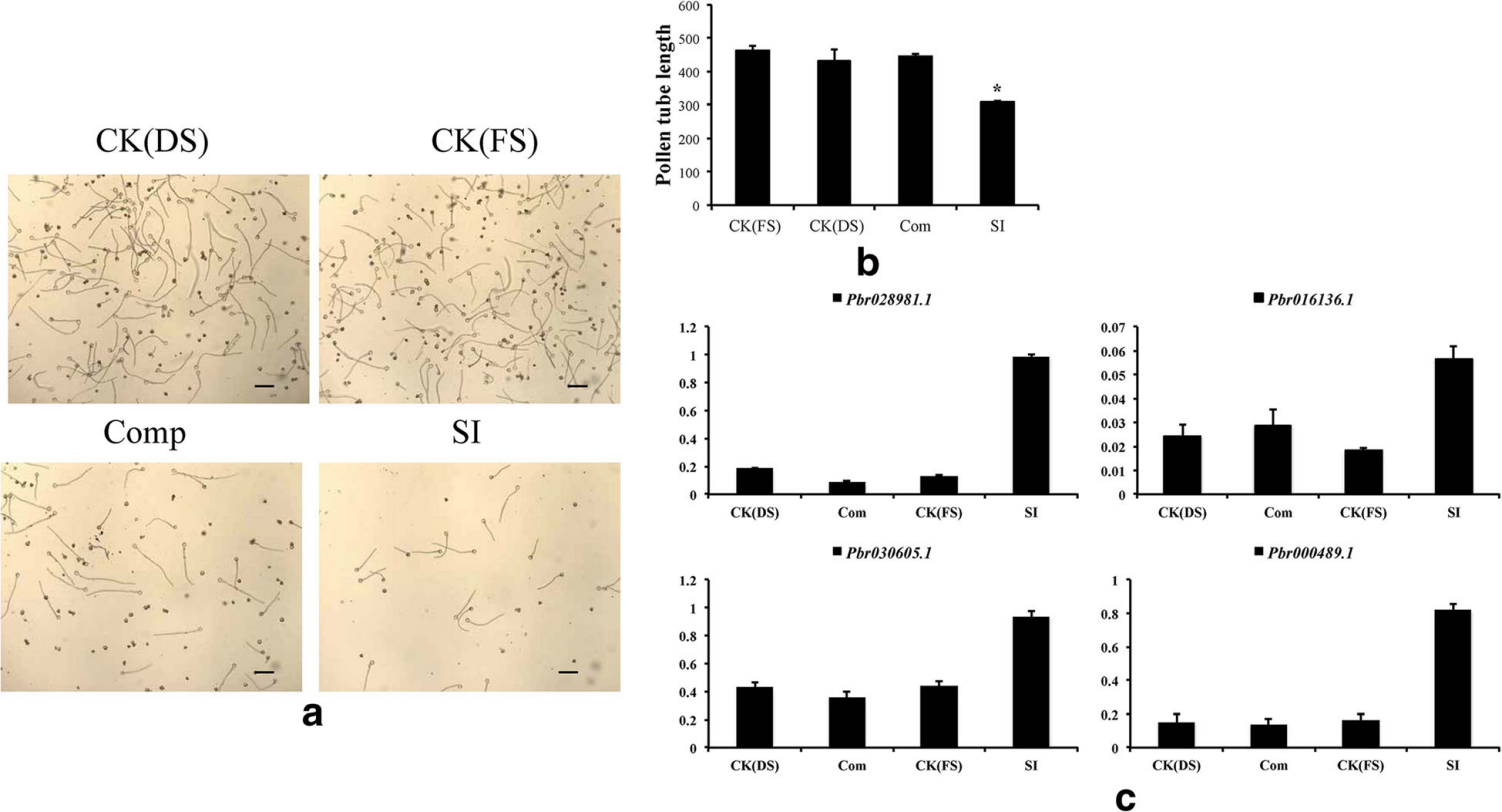
Expression pattern of CRP*s* in self-incompatibility of pollen tubes. **a** Pollen tube treated with self and non-self S-RNase; (**b**) Pollen tube length statistics; (**c**) Relative expression of four CRP genes in pollen tubes with different treatments. CK (DS), normal growing ‘Dangshansuli’ pollen tubes; CK (FS), normal growing ‘Fengshui’ pollen tubes; Comp, ‘Dangshansuli’ pollen tube growth after treatment with ‘Fengshui’ S-RNase; SI, ‘Fengshui’ pollen tube growth after treatment with ‘Fengshui’ S-RNase; CK, pollen tube without S-RNase treatment

To ascertain the expression pattern of 10 CRP genes during self-incompatibility, the expression patterns of the ten CRP genes were investigated in ‘Dangshangsuli’ and ‘Fengshui’ pollens treated with S-RNase extracted from styles of ‘Fengshui’. Based on homology, only four genes were suitable for qPCR analysis. S-RNase proteins extracted from ‘Fengshui’ (final concentration, 0.1 mg/mL) supressed ‘Fengshui’ pollen tube growth significantly but had little effect on the growth of ‘Dangshangsuli’ pollen tubes (Fig. 7a, b). qPCR was used to examine the expression patterns of *Pbr028981.1*, *Pbr016136.1*, *Pbr030605.1* and *Pbr000489.1*. All four of these CRP genes presented a similar expression pattern, with up-regulation in SI pollen tubes. This result is consistent with the RPKM values for up-regulated CRPs in compatible and incompatible pollinated pistils.

## Discussion

### CRPs with or without signal peptides were retained in a biased model

A total of 9556 CRPs from 12 species were identified in this analysis. We explored the classification, organisation, collinearity, evolution and expression patterns of these peptides. CRPs with signal peptides comprised the majority of CRP family members in angiosperm plants, except in green algae. Several rounds of WGD events occurred during the formation of angiosperm plants, and the CRP family expanded in an explosive way, especially those with a signal peptide. However, a limited number of CRPs without signal peptides also underwent gene duplications, although their duplication types were varied. Classification results suggest that CRPs without signal peptides were generally not retained after gene duplication. Classic evolutionary theory predicts that the accumulation of deleterious mutations is more frequent than advantageous mutations in duplicates that lose their function or eventually become pseudo genes [44]. We propose that duplicated CRPs without signal peptides might involve full genetic redundancy that leads to rapid accumulation of deleterious mutations in duplicated genes. Further reverse genetic analyses of sequence homologies and overlapping expression patterns should be performed to verify this hypothesis.

The large number of duplicated CRPs with signal peptides retained in angiosperms may result from partial or unequal genetic redundancy that preserves a stale evolutionary stage. Allowing for a number of divergent subfamilies, neofunctionalisation and subfunctionalisation maintained by unequal genetic redundancy may account for the expansion of CRPs with signal peptides. The 1281 positively selected CRP genes identified here also demonstrate that neofunctionalisation is common during the evolution of CRP genes. According to the gene balance model, duplicates retained after a WGD are more likely to be functionally connected. Together, these observations suggest that CRPs with signal peptides function in a concerted way, as in signal transduction in reproductive organs and plant–bacteria symbiosis.

### CRP gene clusters

Clustered genes are usually formed and maintained by some evolutionary mechanism. Genes in clusters may be involved in a common metabolic pathway in which the gene products function as enzymes; signalling cascade gene products may interact with each other [45, 46]. Selective pressure is thought to promote gene clustering. Coordinated gene expression may be the most common driving force behind gene cluster formation, with tandem duplication of genes serving as another such mechanism [47].

To determine the mechanism underlying CRP gene clustering, we analysed the RNA-seq expression data and duplication types in pear. Of the 15 clusters with more than two clustered genes, eight clustered gene pairs were found to be involved in the coordinated expression of pear pollen development and two in pear fruit development (Table 4). Tandem duplicated CRP genes were more frequent in clustered genes than in CRP genes in pear (Additional file 13: Figure S9). This finding suggests that tandem duplication influenced CRP gene clustering during gene duplication events and that coordinated expression also played an important role in the formation of clustered genes.

**Table 4:** Coordinated expression of clustered CRP genes

RNA-seq dataset	ClusterID	Gene pairs	*| r |*	
Pear pollen development	Cluster 1	Pbr013206.1	Pbr013205.1	0.999**
		Pbr013206.1	Pbr013203.1	0.978**
		Pbr013205.1	Pbr013203.1	0.986**
	Cluster 2	Pbr012199.1	Pbr012200.1	0.981**
	Cluster 3	Pbr014635.1	Pbr014630.1	1.000**
		Pbr014633.1	Pbr014630.1	0.971*
	Cluster 4	Pbr028981.1	Pbr028984.1	1.000**
	Cluster 5	Pbr008944.1	Pbr008945.1	0.983*
	Cluster 6	Pbr031925.1	Pbr031926.1	0.967*
		Pbr031925.1	Pbr031929.1	0.994**
		Pbr031925.1	Pbr031927.1	0.982*
		Pbr031926.1	Pbr031927.1	0.973*
		Pbr031926.1	Pbr031929.1	0.990*
		Pbr031927.1	Pbr031929.1	0.984*
	Cluster 7	Pbr034678.1	Pbr034676.1	0.997**
		Pbr034677.1	Pbr034679.1	0.951*
		Pbr034677.1	Pbr034678.1	0.960*
	Cluster 8	Pbr031119.1	Pbr031118.1	0.991**
Pear fruit development	Cluster 9	Pbr014635.1	Pbr014634.1	0.932**
	Cluster 10	Pbr022002.1	Pbr022000.1	0.974**

r, correlation of gene pairs (absolute value); expression data for each gene represent two RNA-seq trials; *P < 0.05, significant; **P < 0.01, extremely significant

### Expansion of the CRP family was not always enriched by WGD or segmental duplication

In most angiosperm plants, CRP genes retained after WGD and fractionation comprise the majority of all duplicated CRP genes. The species-specific Ks peaks for CRP genes suggest that CRP genes in some species, such as pear, black cottonwood, maize and sorghum, were retained after a WGD event (Fig. 4a). However, CRP genes from a WGD or fraction do not always come from a WGD event, although some species experienced one or more such events. Soybean and cabbage contain a large number of CRP genes from a WGD and have experienced a recent WGD, yet they still had no obvious peak during the occurrence of a recent WGD. Thus, we concluded that CRP genes from a WGD in these species came from several rounds of WGD events and segmental duplications.

Unlike angiosperms, which experienced more than one WGD event, more maize and cabbage CRP genes were observed to have arisen from a single gene duplication than from a WGD or segmental duplication, even though they also have undergone a recent WGD. However, grape also experienced only a gamma event yet contains a number of CRP genes from WGD events [48]; the Ks peak for the CRP genes is out of the occurrence range of the gamma event. This observation suggests that duplicated CRPs in different species evolve differently. While most CRPs were retained after a WGD or segmental duplication, in some species, CRPs from a WGD or segmental duplication underwent further gene movement or transposition.

### CRPs in the grass family are more likely to acquire new functions

Genome-wide scans for positive selection between species are useful for investigating inter-species divergence. Our work identified 1281 positively selected genes, most of which belonged to sorghum and maize (Fig. 3c). Previous analyses revealed a polymorphism pattern in maize and its wild progenitor teosinte (*Z. mays* ssp. *parviglumis*) [49]. Artificial selection is considered to have positively selected 1200–1400 genes in maize. However, our work found that 619 CRP genes in maize fell into the ‘positive selection’ category, while 528 CRP genes of this category were identified in sorghum. Despite the presence of CRP genes, genes for C4 enzymes, especially carbonic anhydrase, indicate the formation of new independent genes in C3 plants.

As the Ks peak of positively selected genes indicates, artificial selection by humans over the last 7500 years does not seem to be the factor that generated of the explosive expansion of CRPs that were positively selected in maize and sorghum (Fig. 4b). These results suggest that positively selected CRPs were generated by the shared maize and sorghum ancestor about 11 million years ago during two rounds of WGD and segmental duplications (Additional file 6: Figure S3a and Fig. 4).

Positive selection usually affects gene function via expression pattern. In *Drosophila*, *Zeus* under positive selection reshapes the global sex biased gene expression [50]. After gene duplication, the functional divergence of Venus flytrap module that driven by positive selection occurs [51]. Our analysis suggests that three pear CRPs were positive selected by their protein structure variations and that the expression pattern in pollen tube development also diverged. Recent reports of Faster-Z evolution in birds suggest that positive selection acts more on gene expression than sequence [52]. In our analysis, both CRP expression and protein sequences in pear pollen development were affected by adaptive evolution. However, details of the function of the three CRPs in pear and their homologues in other species are still unknown.

### Evolutionary rates and expression divergence of CRP genes generated by different duplication models

Although we compared the evolutionary rates between CRP genes in different subfamilies using different duplication models, an insufficient number of genes in some categories restricted our analysis. We found that CRP genes retained after a WGD and segmental duplication had a faster evolutionary rate than those retained from a single gene duplication (Fig. 5). The dispersed duplication model might yield a higher evolutionary rate than did other duplication models (Additional file 7: Figure S4). However, it is difficult to estimate the evolutionary rate of CRP genes that underwent more than one duplication event. For example, one gene can be duplicated by a WGD event and transposed by transposons to another location on the chromosome. After transposition, the expression patterns might change, and this gene could be subjected to further selective pressure. Using a sufficient number of genes and subfamilies for analysis using different duplication models can overcome this problem. Divergence of CRP gene expression was also affected by the gene duplication type, according to two pear transcriptome datasets. Our results show that different expression libraries may present different correlations between gene duplication types, suggesting that the fates of the different CRP duplication types varied between tissues. The different duplication types also differed with respect to expression divergence in each library, suggesting that different duplication mechanisms drive expression divergence.

### Pear CRPs participate in self-compatibility of pollen tubes

S-RNase secreted by the style is released into growing pollen tubes and inhibits self-pollen tube growth. Our results of RNA-seq and qPCR, both in vivo and in vitro, show that *CRPs* are up-regulated during self-incompatibility. We also found that the up-regulated CRPs belong to two distinct subfamilies that share some common arrangements of Cys residues (Fig. 6a). This observation suggests that CRPs play a variety of roles in S-RNase–based self-incompatibility. How these CRPs function in self-incompatibility is an interesting question that warrants further investigation.

## Conclusion

To investigate the formation and genetic features of divergent CRPs in plants, our analysis of 12 species focuses on the expansion pattern, evolution rate, divergence time and expression divergence of 9556 CRPs. We found that CRPs have several characteristics: 1) Most duplicated CRPs arose from WGD or segmental duplication; 2) The distribution of CRPs in some species is clustered; 3) Positively selected CRPs were the majority in maize and sorghum; 4) In pear, CRPs responded to SI via gene expression. Considering the large number of CRP genes in plant genomes, the characteristics of these genes and the function of their products warrant exploration in further studies.

## Additional files

Additional file 1: Table S4. Primers used to amplify CRPs in qPCR. (XLSX 50 kb)

Additional file 2: Table S1. Genomic CRP information for all 12 species. S, secretory pathway; C, chloroplast; M, mitochondrial; −, no signal peptide or an unknown type of signal peptide. (XLSX 1744 kb)

Additional file 3: Figure S1. Conserved cysteine motifs in three large subfamilies: Lipid Transfer Protein, Pollen Ole I and DEFL. (PDF 20131 kb)

Additional file 4: Table S2. Collinear gene pairs. Collinearity analysis was carried out using MCScanX and screened for E value <0.01. (XLSX 147 kb)

Additional file 5: Figure S2. CRP distribution in cabbage, soybean, black cottonwood, tomato, maize, sorghum and grape. Dashed green line, CRPs in chromosomes and their genomic locations (above the chromosomes). Blue lines across the chromosomes, collinear relationship between different CRPs. Numbers in the parentheses, collinear gene pair number. (TIFF 12790 kb)

Additional file 6: Figure S3. Relationship between WGD events and the number of duplicated CRP genes. a) Brief phylogenetic tree of sequenced species; b) Duplication type of CRP genes from 12 species. WGD data were obtained from the Plant Genome Duplication Database [53]. (PDF 285 kb)

Additional file 7: Figure S4. Comparison of Ks values for CRP genes. a) Comparison of Ks values for CRP genes from 11 angiosperm plants; b) Comparison of Ks values between positively and negatively selected genes. (PDF 191 kb)

Additional file 8: Figure S5. Comparison of evolutionary rates between four duplication models in strawberry, mei and peach. (PDF 185 kb)

Additional file 9: Table S3. Sites of positively selected pear CRP genes as estimated by BEB analysis under the M8 model. (XLSX 52 kb)

Additional file 10: Figure S6. Expression pattern of CRP genes during fruit development. S1–S6 indicate stages of fruit development at 15, 36, 80, 110, 145 and 167 days after flowering. (PDF 586 kb)

Additional file 11: Figure S7. Expression pattern of CRP genes for different pollen and pollen tube development stages. HP, hydrated pollen; MP, mature pollen; PT, hydrated pollen tube; SPT, stopped pollen tube. (PDF 878 kb)

Additional file 12: Figure S8. Comparison of expression divergence between CRPs with different types of duplication. a) Comparison of distribution and level of expression divergence of CRPs expressed in pollen and pollen tube development. b) Comparison of distribution and level of expression divergence of CRPs expressed during fruit development. Expression divergence was measured by 1-∂, where ∂ is the correlation between expression profiles. (PDF 2530 kb)

Additional file 13: Figure S9. Percentage of each duplication type in clustered CRP genes in pear. a) Percentage of each duplication type among clustered pear CRP genes; b) Percentage of each duplication type among total pear CRP genes. (TIF 198 kb)

## Abbreviations

CA: Carbonic anhydrase
CRP: Cysteine-rich peptide
DEFL: Defensin-like sequences
LTR: Long terminal repeat retrotransposons
RALF: Rapid alkalinisation factor
SI: Self-incompatibility
WGD: Whole genome Duplication
