# Supplementary material for: Expansion and evolutionary patterns of cysteine-rich peptides in plants
Source: BMC Genomics. 2017 Aug 14;18:610. doi: 10.1186/s12864-017-3948-3 (PMC5557327; doi:10.1186/s12864-017-3948-3)
Supplement: Supplementary file 3 — Conserved cysteine motifs in three large subfamilies: Lipid Transfer Protein, Pollen Ole I and DEFL. (PDF 20131 kb) [file 12864_2017_3948_MOESM3_ESM.pdf]

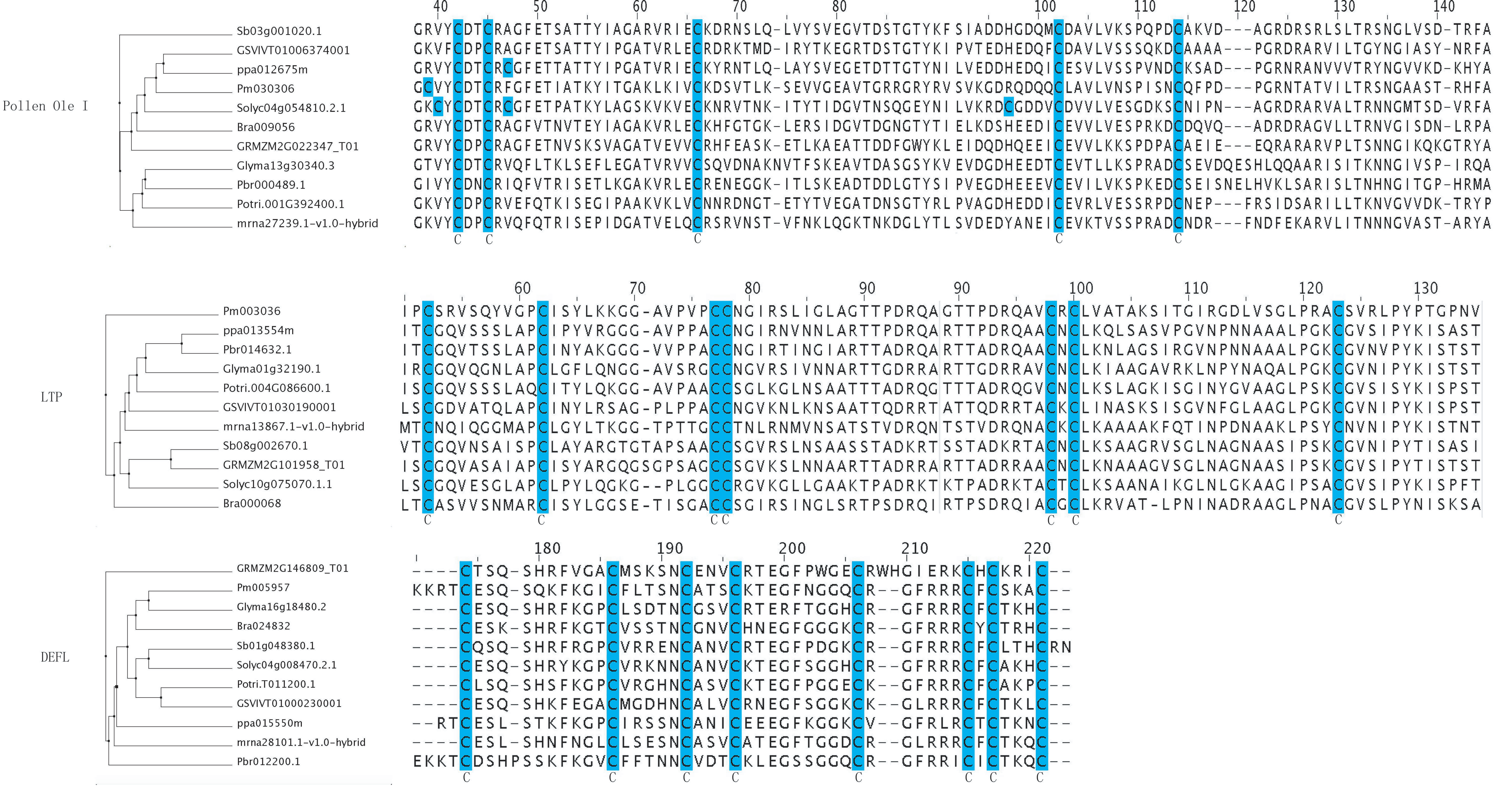

Pollen Ole I
Sb03g001020.1 GRVYCDTCRAGFETSATTYIAGARVRIECKDRNSLQ-LVYSVEGVTDSTGTYKFSIADDHGDQMCDAVLVKSPQPDCAKVD---AGRDRSRLSLTRSNGLVSD-TRFA
GSVIVT01006374001 GKVFCDPCRAGFETSATTYIPGATVRLECRDRKTMD-IRYTKEGRTDSTGTYKIPVTEDHEDQFCDAVLVSSSQKDCAAAA---PGRDRARVILTGYNGIASY-NRFA
ppa012675m GRVYCDTCRCGFETTATTYIPGATVRIECKYRNTLQ-LAYSVEGETDTTGTYNILVEDDHEDQICESVLVSSPVNDCKSAD---PGRNRANVVVTRYNGVVKD-KHYA
Pm030306 GCVYCDTCRFGFETIATKYITGAKLKIVCKDSVTLK-SEVVGEAVTGRRGRYRVSVKGDRQDQQCLAVLVNSPISNCQFPD---PGRNTATVILTRSNGAAST-RHFA
Solyc04g054810.2.1 GKCYCDTCRCGFETPATKYLAGSKVKVECKNRVTNK-ITYTIDGVTNSQGEYNILVKRDCGDDVCDVVLVESGDKSCNIPN---AGRDRARVALTRNNGMTSD-VRFA
Bra009056 GRVYCDTCRAGFVTNVTEYIAGAKVRLECKHFGTGK-LERSIDGVTDGNGTYTIELKDSHEEDICEVVLVESPRKDCDQVQ---ADRDRAGVLLTRNVGISDN-LRPA
GRMZM2G022347_T01 GRVYCDPCRAGFETNVSKSVAGATVEVVCRHFEASK-ETLKAEATTDDFGWYKLEIDQDHQEEICEVVLKKSPDPACAEIE---EQRARARVPLTSNNGIKQKGTRYA
Glyma13g30340.3 GTVYCDTCRVQFLTKLSEFLEGATVRVVCSQVDNAKNVTFSKEAVTDASGSYKVEVDGDHEEDTCEVTLLKSPRADCSEVDQESHLQQAARISITKNNGIVSP-IRQA
Pbr000489.1 GIVYCDNCRIQFVTRISETLKGAKVRLECRENEGGK-ITLSKEADTDDLGTYSIPVEGDHEEEVCEVILVKSPKEDCSEISNELHVKLSARISLTNHNGITGP-HRMA
Potri.001G392400.1 GKVYCDPCRVEFQTKISEGIPAAKVKLVCNNRDNGT-ETYTVEGATDNSGTYRLPVAGDHEDDICEVRLVESSRPDCNEP---FRSIDSARILLTKNVGVVDK-TRYP
mrna27239.1-v1.0-hybrid GKVYCDPCRVQFQTRISEPIDGATVELQCRSRVNST-VFNKLQGKTNKDGLYTLSVDEDYANEICEVKTVSSPRADCNDR---FNDFEKARVLITNNNGVAST-ARYA
LTP
Pm003036 IPCSRVSQYVGPCISYLKKGG-AVPVPCCNGIRSLIGLAGTTPDRQAGTTPDRQAVCRCLVATAKSITGIRGDLVSGLPRACSVRLPYPTGPNV
ppa013554m ITCGQVSSSLAPCIPYVRGGG-AVPPACCNGIRNVNNLARTTPDRQARTTPDRQAACNCLKQLSASVPGVNPNNAAALPGKCGVSIPYKISAST
Pbr014632.1 ITCGQVTSSLAPCINYAKGGG-VVPPACCNGIRTINGIARTTADRQARTTADRQAACNCLKNLAGSIRGVNPNNAAALPGKCGVNVPYKISTST
Glyma01g32190.1 IRCGQVQGNLAPCLGFLQNGG-AVSRGCCNGVRSIVNNARTTGDRRARTTGDRRAVCNCLKIAAGAVRKLNPYNAQALPGKCGVNIPYKISTST
Potri.004G086600.1 ISCGQVSSSLAQCITYLQKGG-AVPAACCSGLKGLNSAATTTADRQGTTTADRQGVCNCLKSLAGKISGINYGVAAGLPSKCGVSISYKISPST
GSVIVT01030190001 LSCGDVATQLAPCINYLRSAG-PLPPACCNGVKNLKNSAATTQDRRTATTQDRRTACKCLINASKSISGVNFGLAAGLPGKCGVNIPYKISPST
mrna13867.1-v1.0-hybrid MTCNQIQGGMAPCLGYLTKGG-TPTTGCCTNLRNMVNSATSTVDRQNTSTVDRQNACKCLKAAAAKFQTINPDNAAKLPSYCNVNIPYKISTNT
Sb08g002670.1 VTCGQVNSAISPCLAYARGTGTAPSAACCSGVRSLNSAASSTADKRTSSTADKRTACNCLKSAAGRVSGLNAGNAASIPSKCGVNIPYTISASI
GRMZM2G101958_T01 ISCGQVASAIAPCISYARGQGSGPSAGCCSGVKSLNNAARTTADRRARTTADRRAACNCLKNAAAGVSGLNAGNAASIPSKCGVSIPYTISTST
Solyc10g075070.1.1 LSCGQVESGLAPCLPYLQGKG--PLGGCCRGVKGLLGAAKTPADRKTKTPADRKTACTCLKSAANAIKGLNLGKAAGIPSACGVSIPYKISPFT
Bra000068 LTCASVVSNMARCISYLGGSE-TISGACCSGIRSINGLSRTPSDRQIRTPSDRQIACGCLKRVAT-LPNINADRAAGLPNACGVSLPYNISKSA
DEFL
GRMZM2G146809_T01 ----CTSQ-SHRFVGACMSKSNCENVCRTEGFPWGECRWHGIERKCHCKRIC--
Pm005957 KKRTCESQ-SQKFKGICFLTSNCATSCKTEGFNGGQCR--GFRRRCFCSKAC--
Glyma16g18480.2 ----CESQ-SHRFKGPCLSDTNCGSVCRTERFTGGHCR--GFRRRCFCTKHC--
Bra024832 ----CESK-SHRFKGTCVSSTNCGNVCHNEGFGGGKCR--GFRRRCYCTRHC--
Sb01g048380.1 ----CQSQ-SHRFRGPCVRRENCANVCRTEGFPDGKCR--GFRRRCFCLTHCRN
Solyc04g008470.2.1 ----CESQ-SHRYKGPCVRKNNCANVCKTEGFSGGHCR--GFRRRCFCAKHC--
Potri.T011200.1 ----CLSQ-SHSFKGPCVRGHNCASVCKTEGFPGGECK--GFRRRCFCAKPC--
GSVIVT01000230001 ----CESQ-SHKFEGACMGDHNCALVCRNEGFSGGKCK--GLRRRCFCTKLC--
ppa015550m --RTCESL-STKFKGPCIRSSNCANICEEEGFKGGKCV--GFRLRCTCTKNC--
mrna28101.1-v1.0-hybrid ----CESL-SHNFNGLCLSESNCASVCATEGFTGGDCR--GLRRRCFCTKQC--
Pbr012200.1 EKKTCDSHPSSKFKGVCFFTNNCVDTCKLEGSSGGQCR--GFRRICICTKQC--
