# Supplementary figures and images for: Expansion and evolutionary patterns of cysteine-rich peptides in plants
Source: BMC Genomics. 2017 Aug 14;18:610. doi: 10.1186/s12864-017-3948-3 (PMC5557327; doi:10.1186/s12864-017-3948-3)

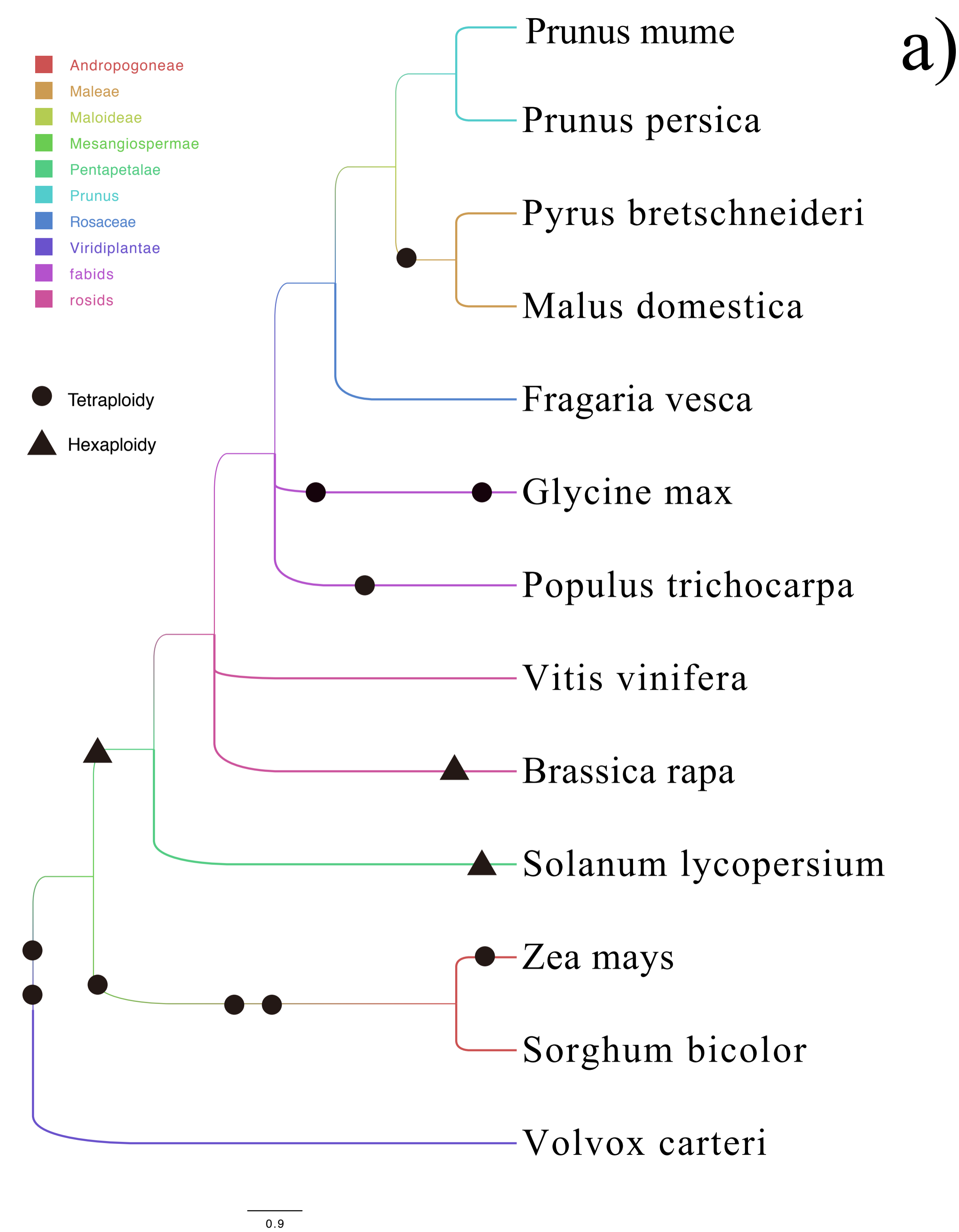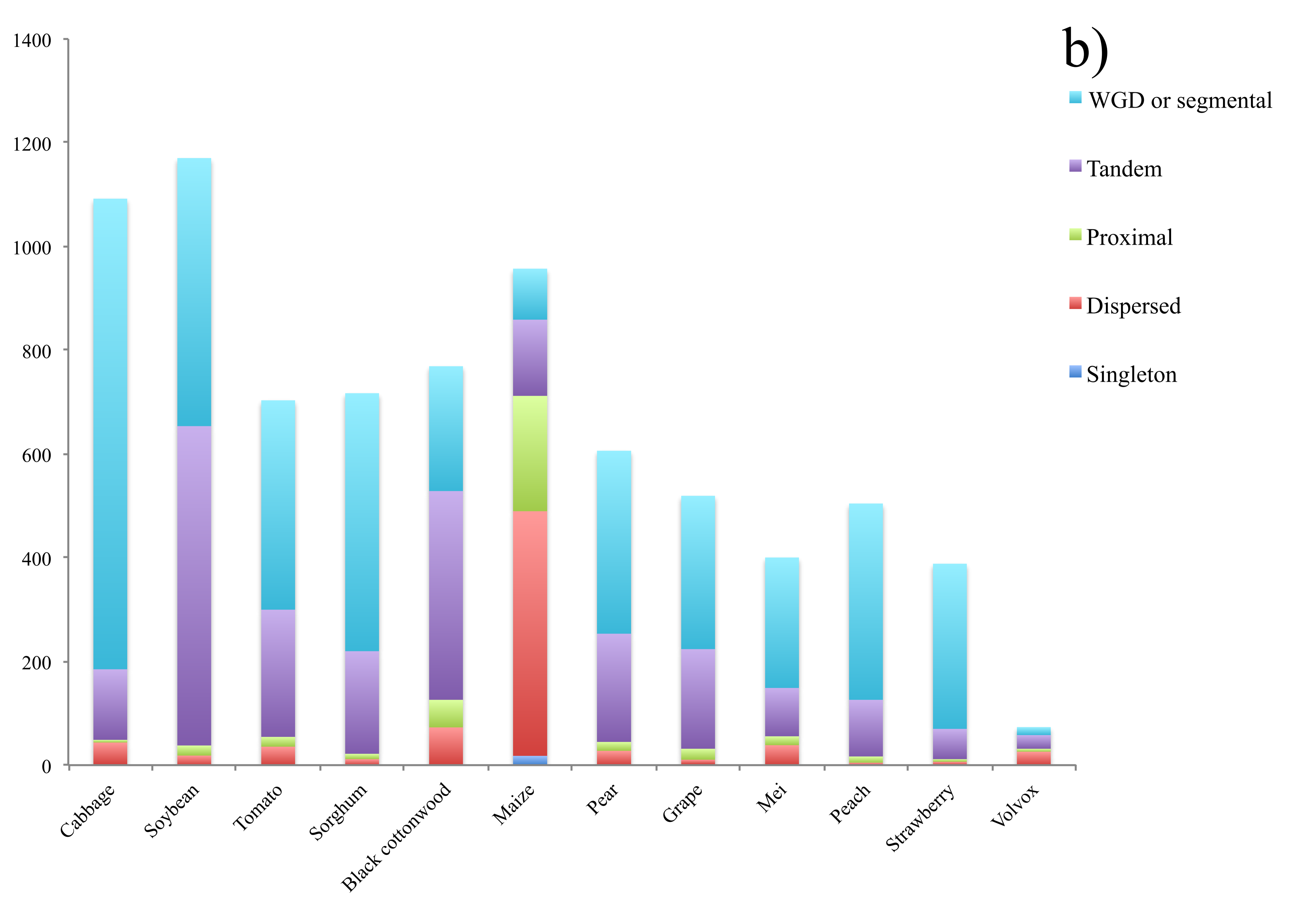

Supplement: Supplementary file 6 — Relationship between WGD events and the number of duplicated CRP genes. a) Brief phylogenetic tree of sequenced species; b) Duplication type of CRP genes from 12 species. WGD data were obtained from the Plant Genome Duplication Database [53]. (PDF 285 kb) [file 12864_2017_3948_MOESM6_ESM.pdf]

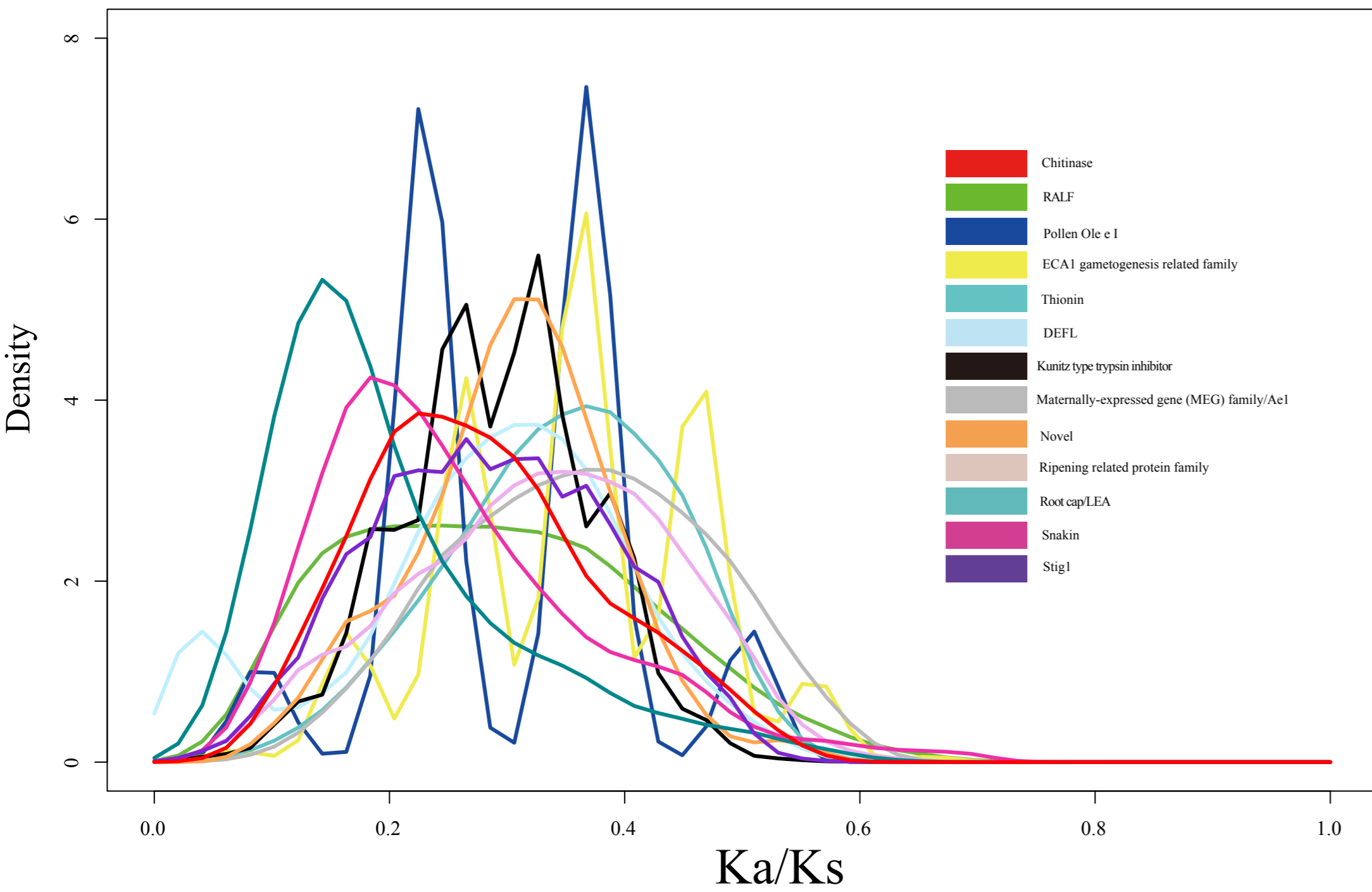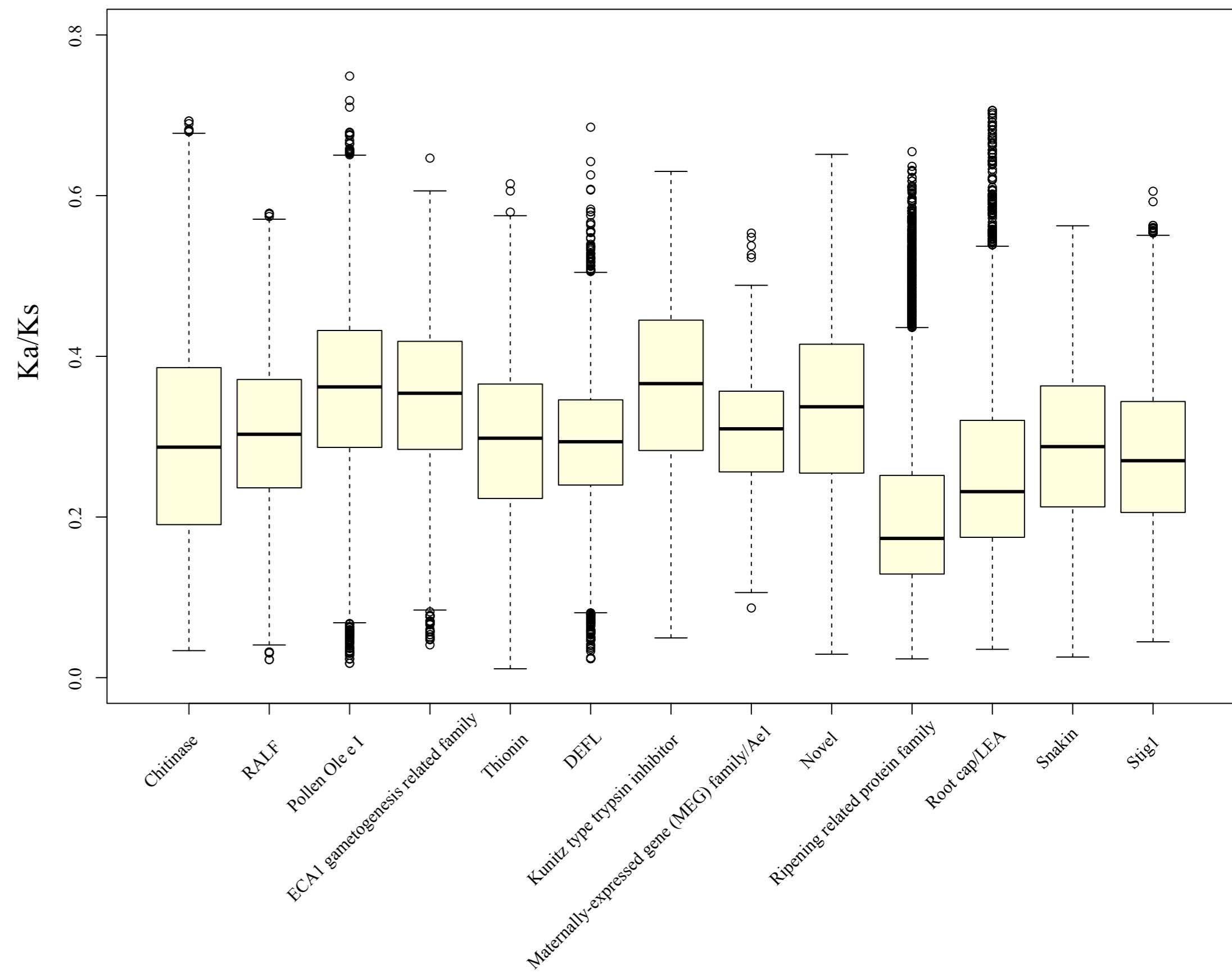

Supplement: Supplementary file 7 — Comparison of Ks values for CRP genes. a) Comparison of Ks values for CRP genes from 11 angiosperm plants; b) Comparison of Ks values between positively and negatively selected genes. (PDF 191 kb) [file 12864_2017_3948_MOESM7_ESM.pdf]

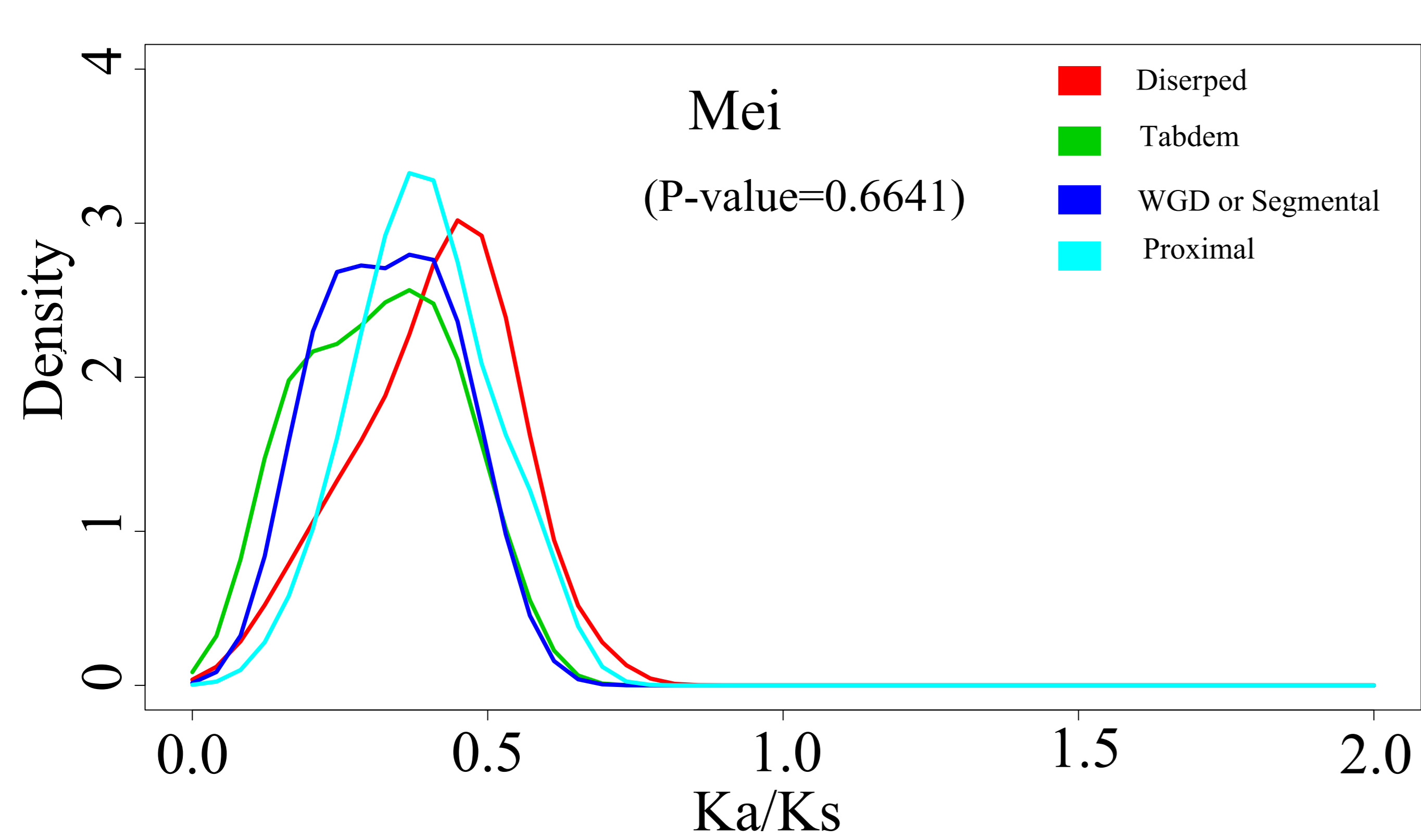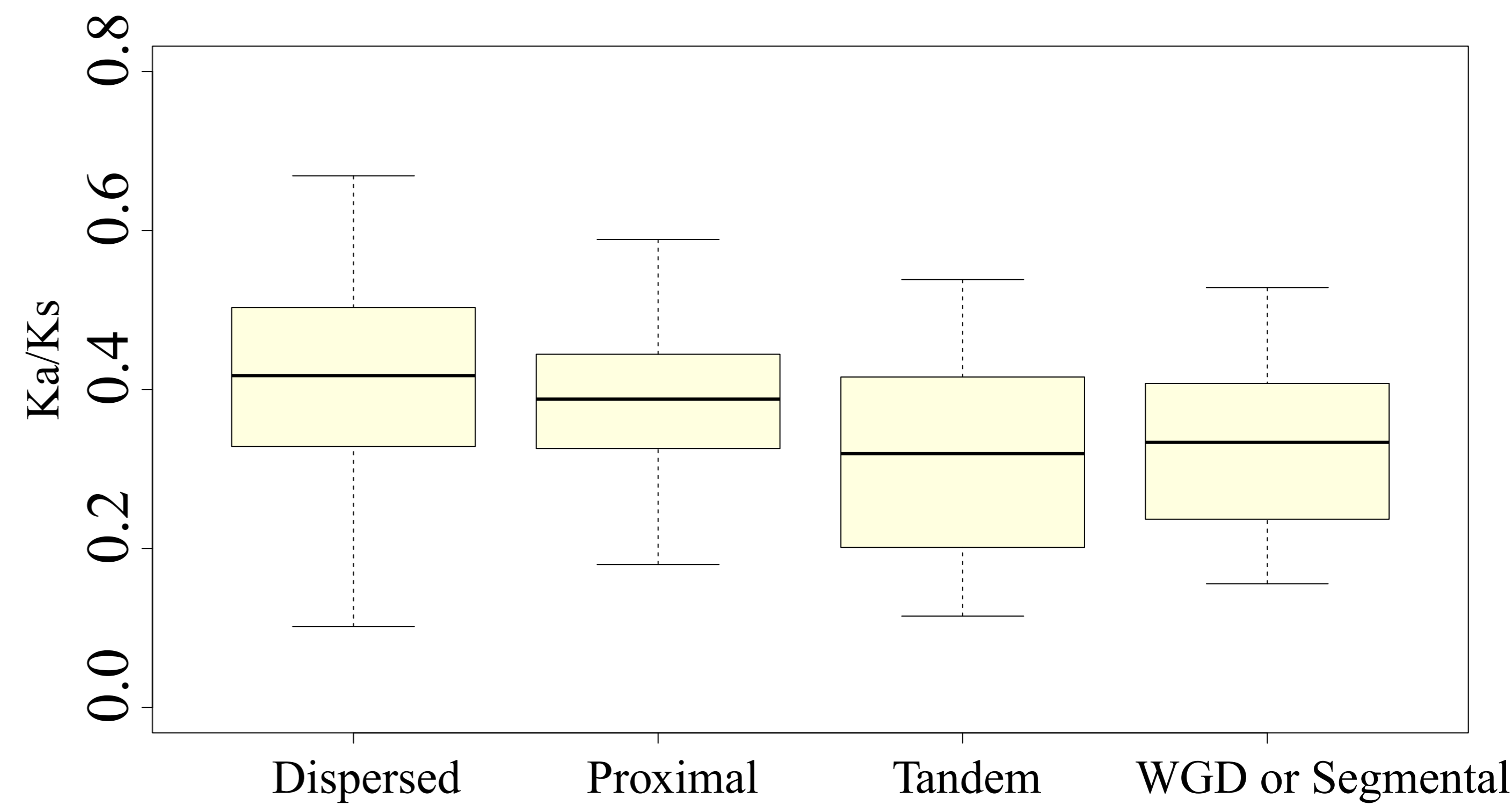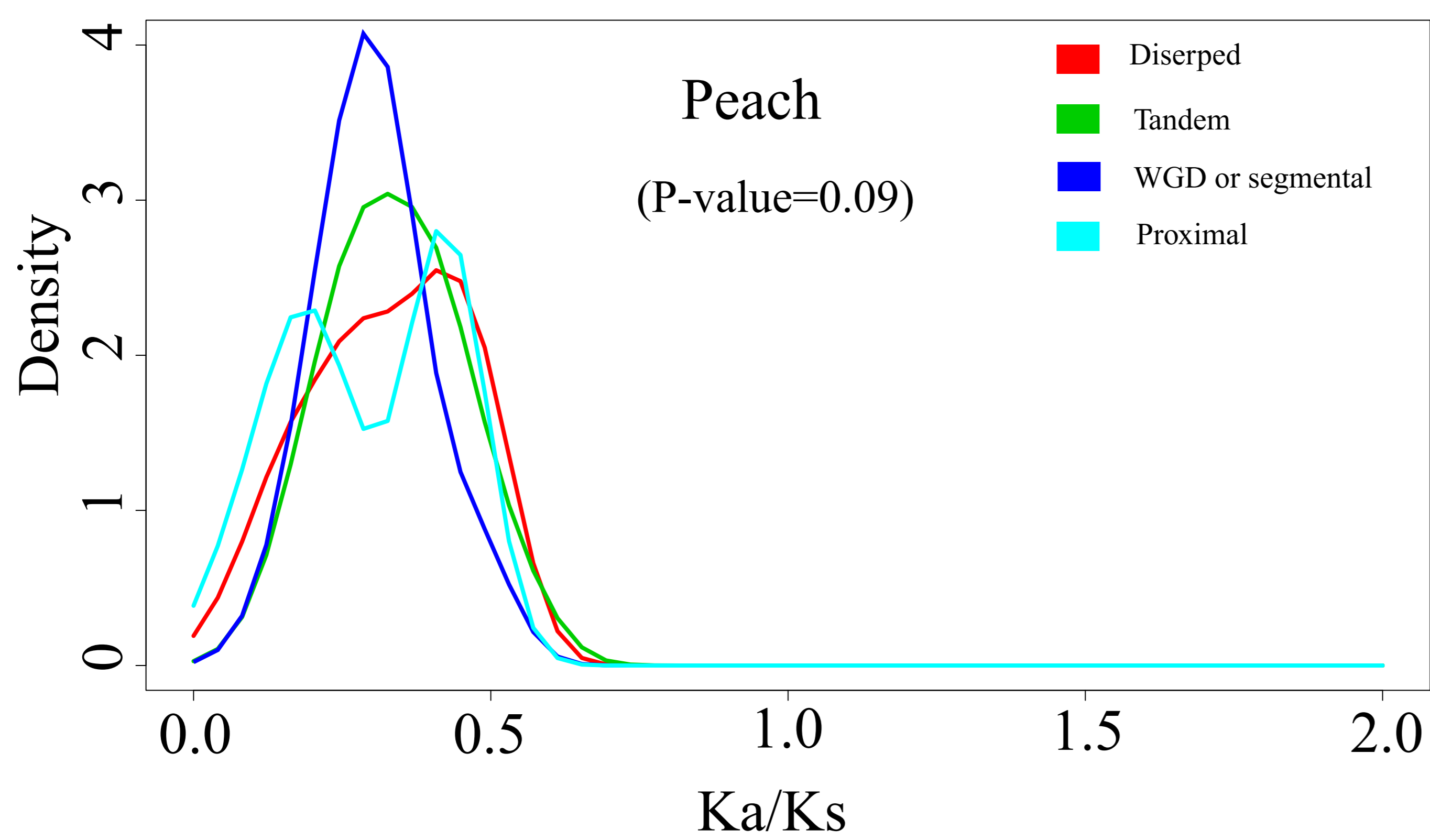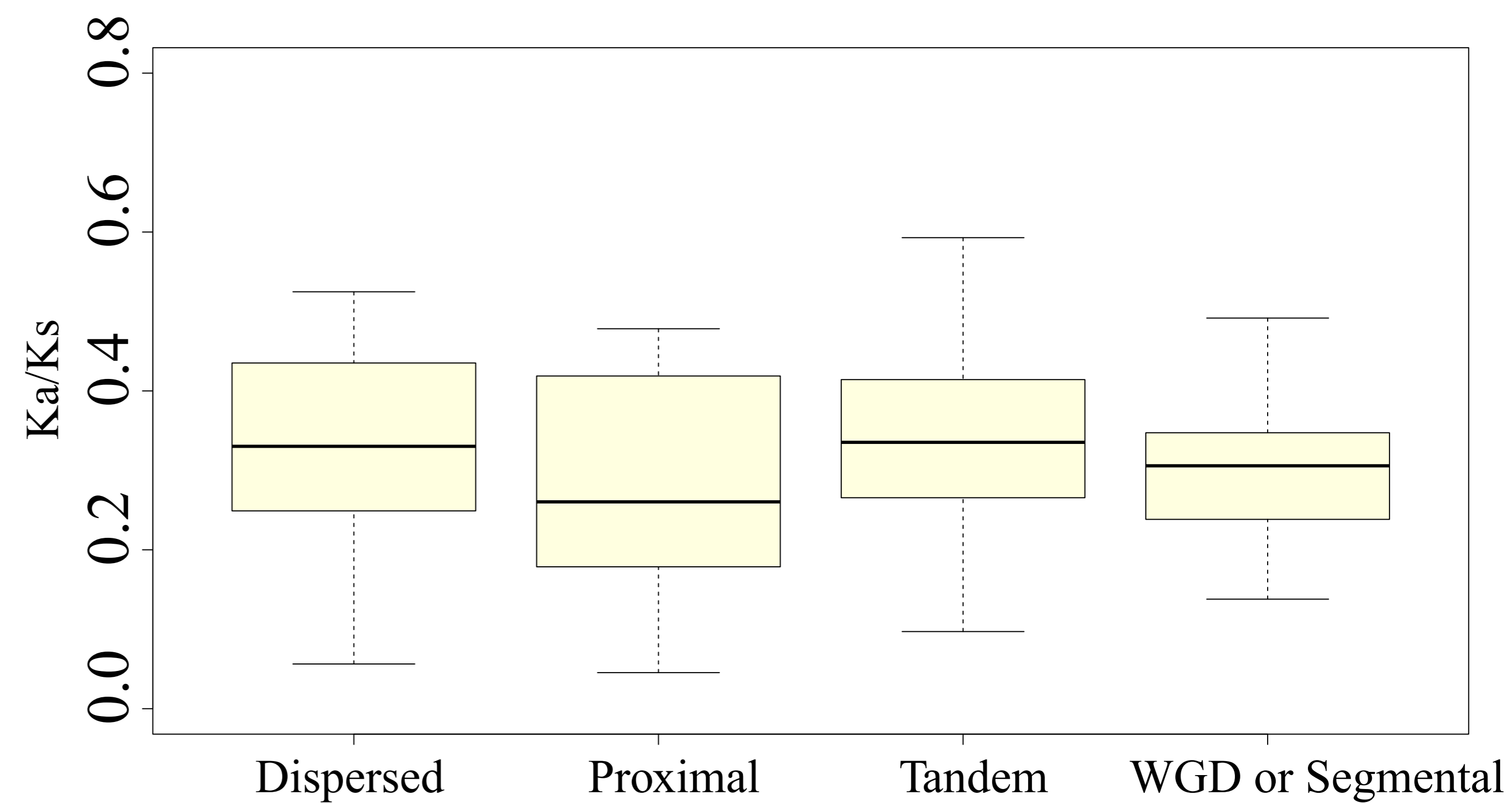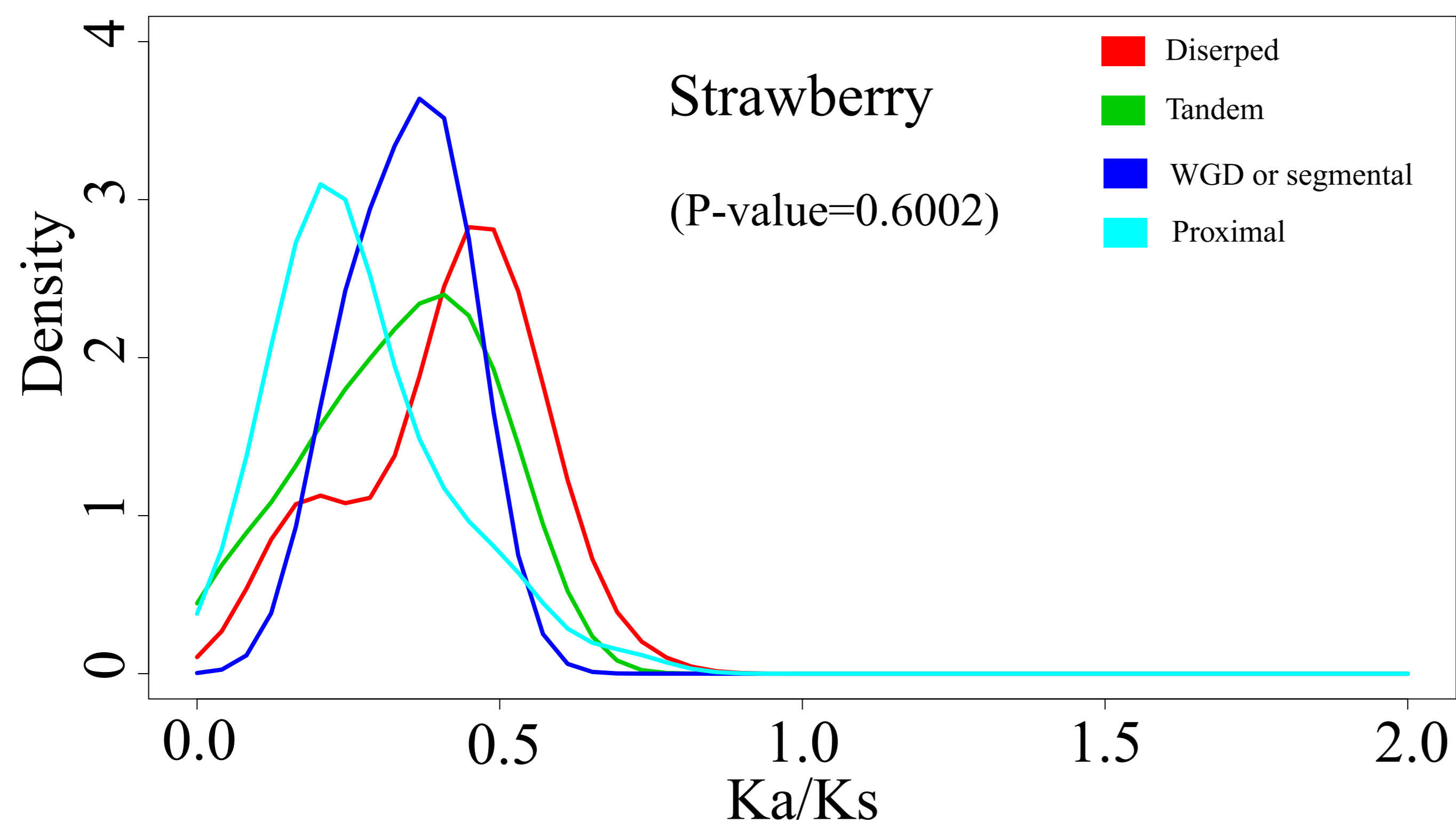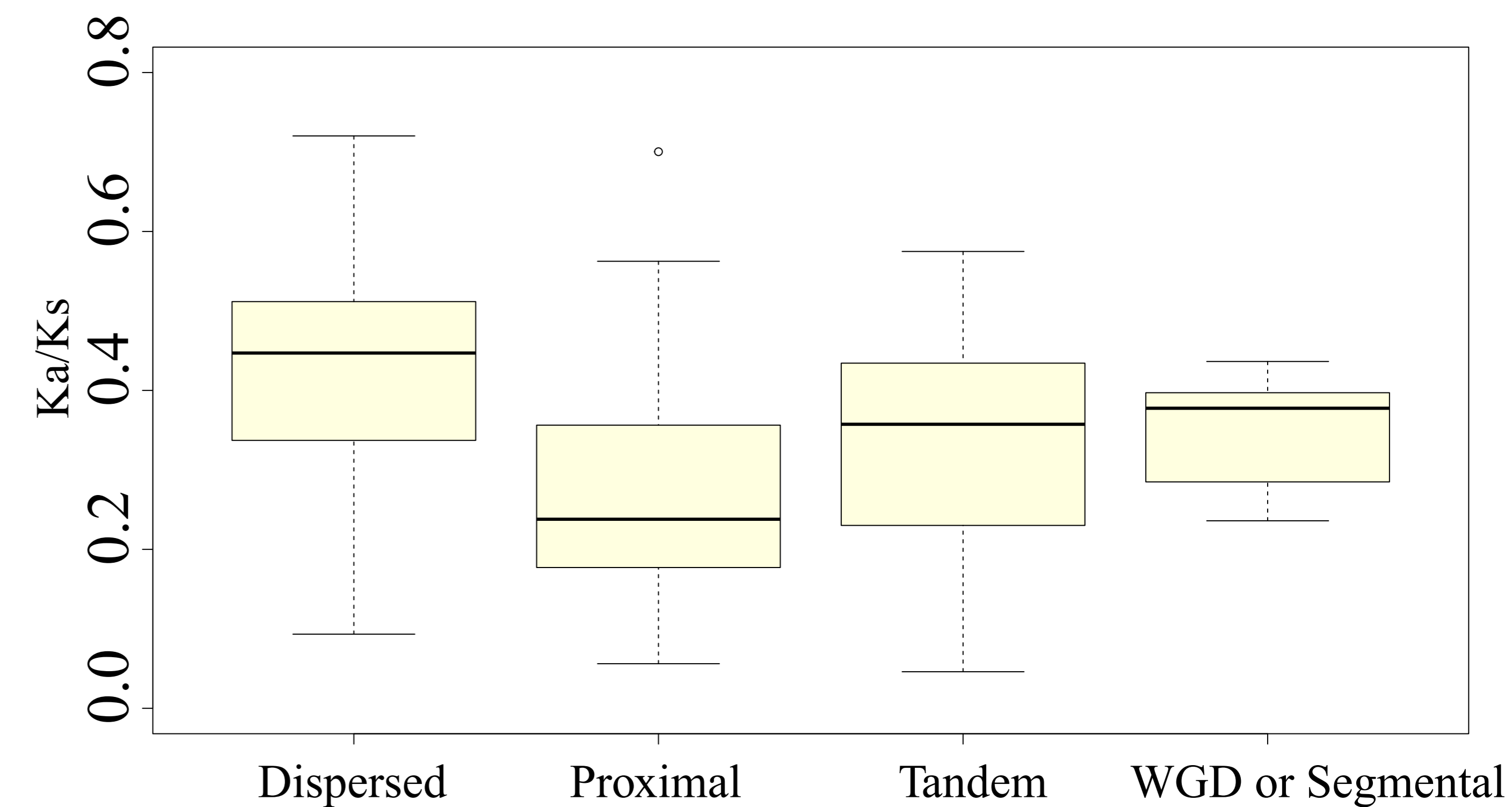

Supplement: Supplementary file 8 — Comparison of evolutionary rates between four duplication models in strawberry, mei and peach. (PDF 185 kb) [file 12864_2017_3948_MOESM8_ESM.pdf]

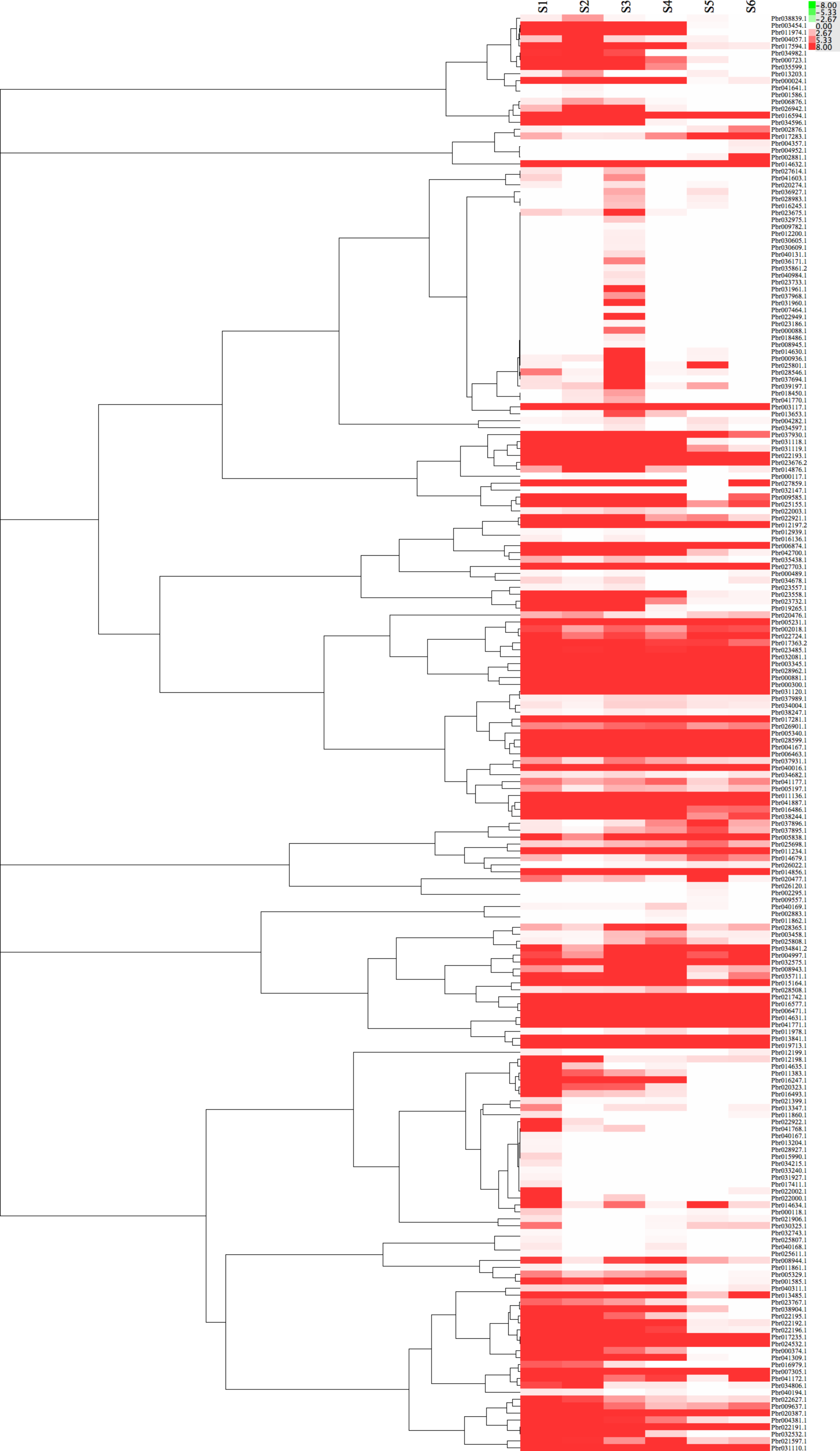

Supplement: Supplementary file 10 — Expression pattern of CRP genes during fruit development. S1–S6 indicate stages of fruit development at 15, 36, 80, 110, 145 and 167 days after flowering. (PDF 586 kb) [file 12864_2017_3948_MOESM10_ESM.pdf]

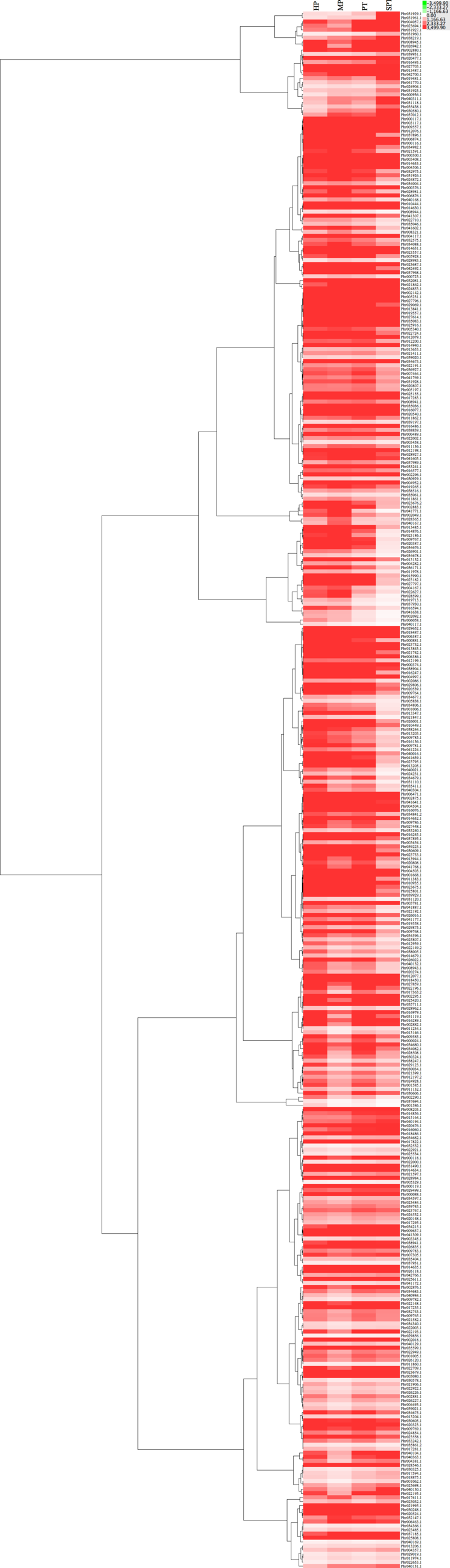

Supplement: Supplementary file 11 — Expression pattern of CRP genes for different pollen and pollen tube development stages. HP, hydrated pollen; MP, mature pollen; PT, hydrated pollen tube; SPT, stopped pollen tube. (PDF 878 kb) [file 12864_2017_3948_MOESM11_ESM.pdf]

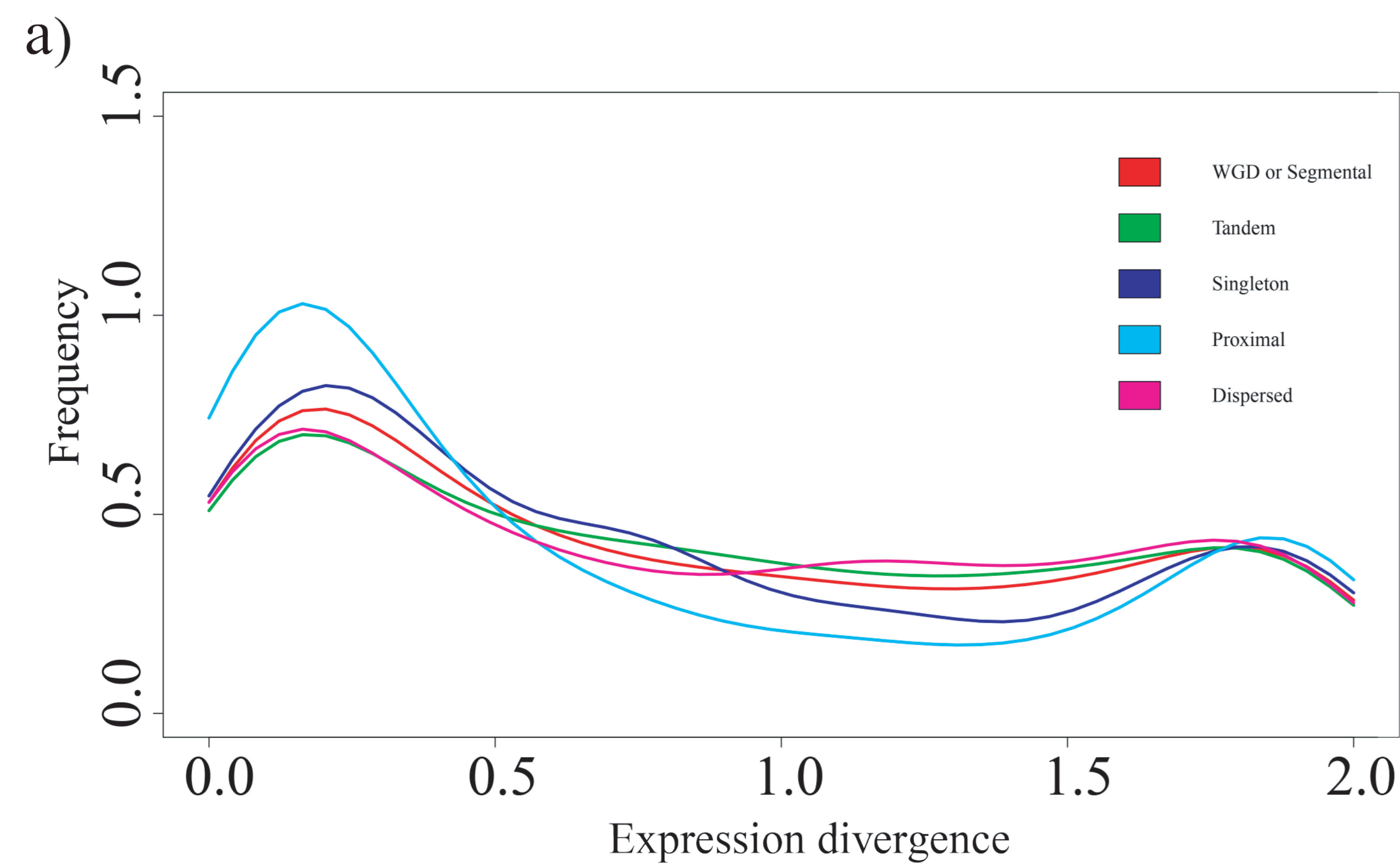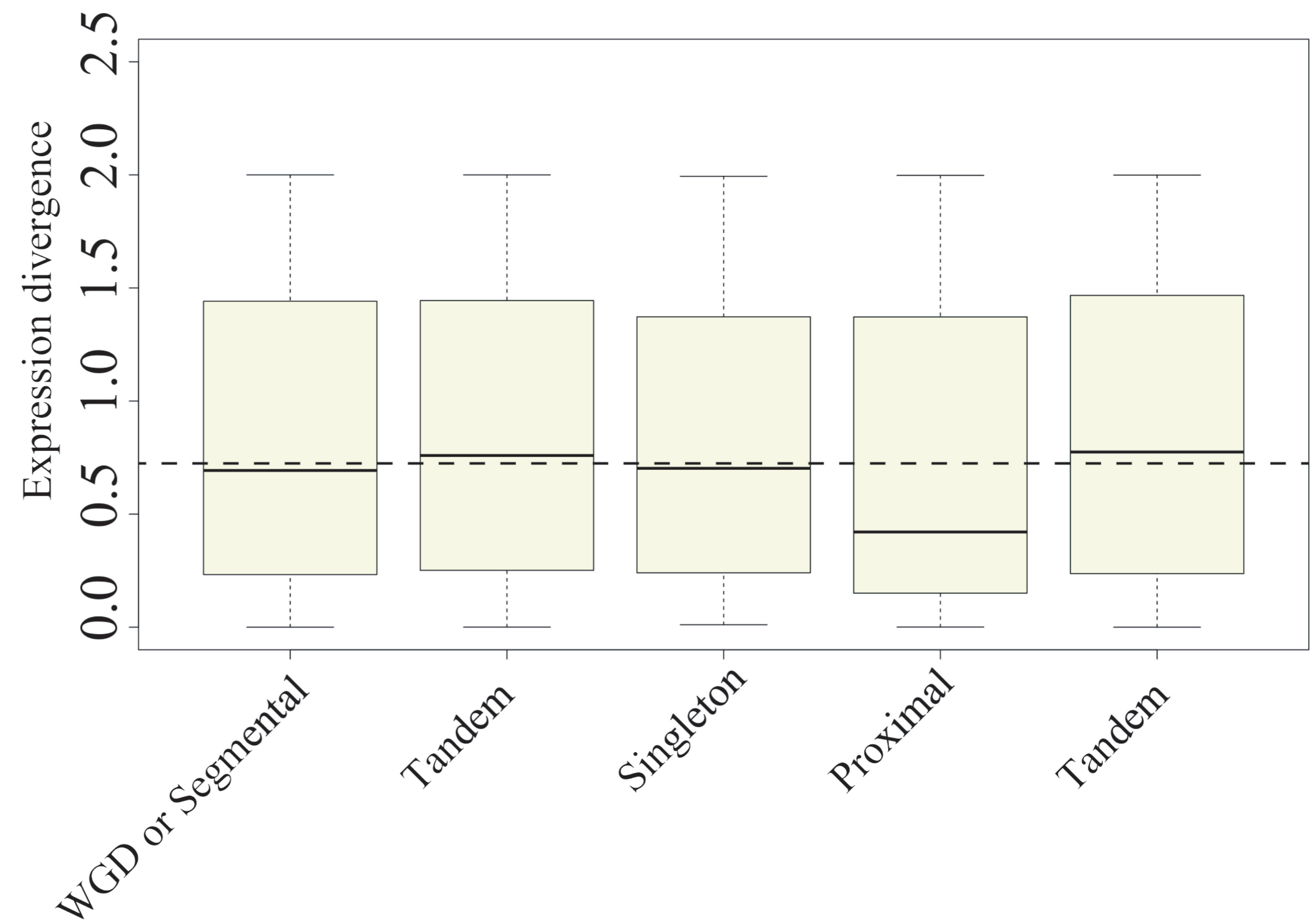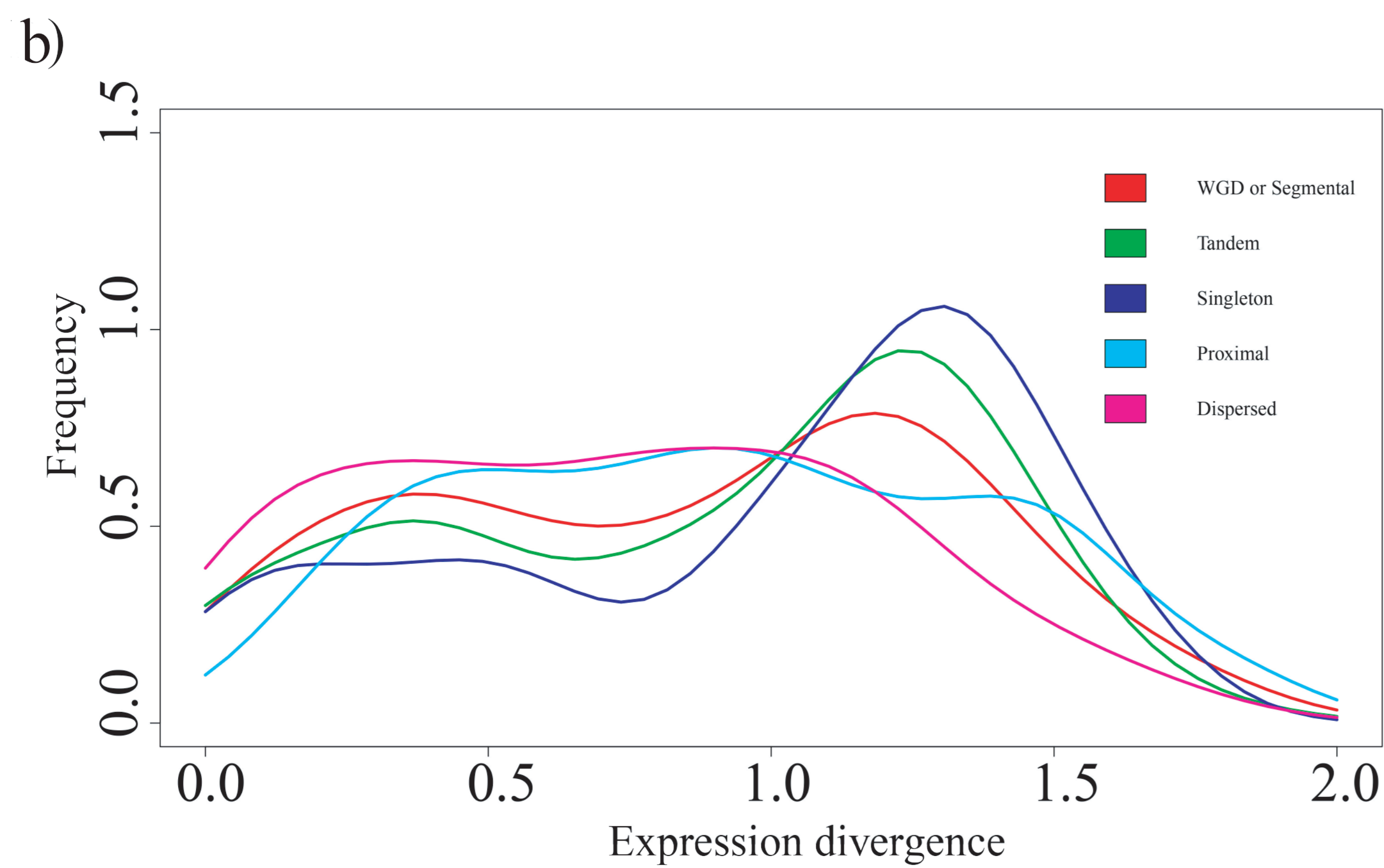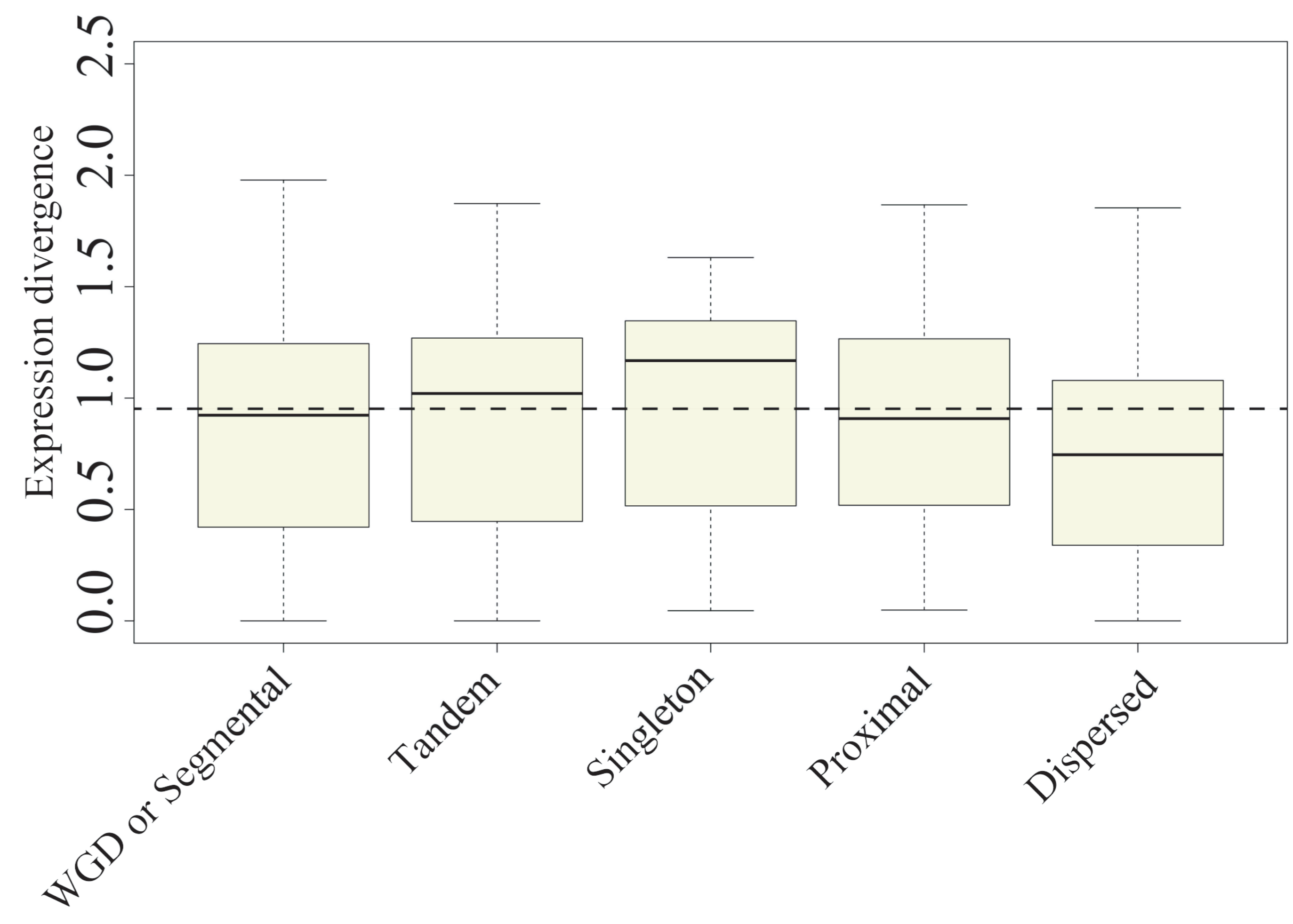

Supplement: Supplementary file 12 — Comparison of expression divergence between CRPs with different types of duplication. a) Comparison of distribution and level of expression divergence of CRPs expressed in pollen and pollen tube development. b) Comparison of distribution and level of expression divergence of CRPs expressed during fruit development. Expression divergence was measured by 1-∂, where ∂ is the correlation between expression profiles. (PDF 2530 kb) [file 12864_2017_3948_MOESM12_ESM.pdf]

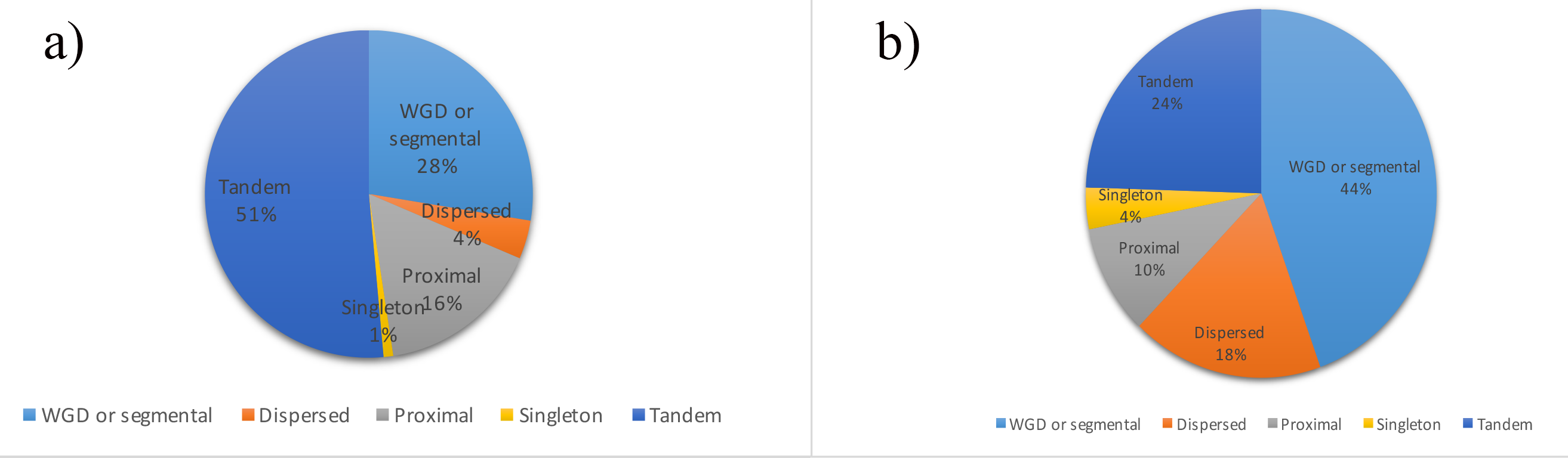

Supplement: Supplementary file 13 — Percentage of each duplication type in clustered CRP genes in pear. a) Percentage of each duplication type among clustered pear CRP genes; b) Percentage of each duplication type among total pear CRP genes. (TIF 198 kb) [file 12864_2017_3948_MOESM13_ESM.tif]
